# Supplementary material for: Robust genome editing via modRNA-based Cas9 or base editor in human pluripotent stem cells
Source: Cell Rep Methods. 2022 Sep 7;2(9):100290. doi: 10.1016/j.crmeth.2022.100290 (PMC9499999; doi:10.1016/j.crmeth.2022.100290)

Cell Reports Methods, Volume *2*

Supplemental information

Robust genome editing via modRNA-based Cas9 or base editor in human pluripotent stem cells

Tahir Haideri, Alessandro Howells, Yuqian Jiang, Jian Yang, Xiaoping Bao, and Xiaojun Lance Lian

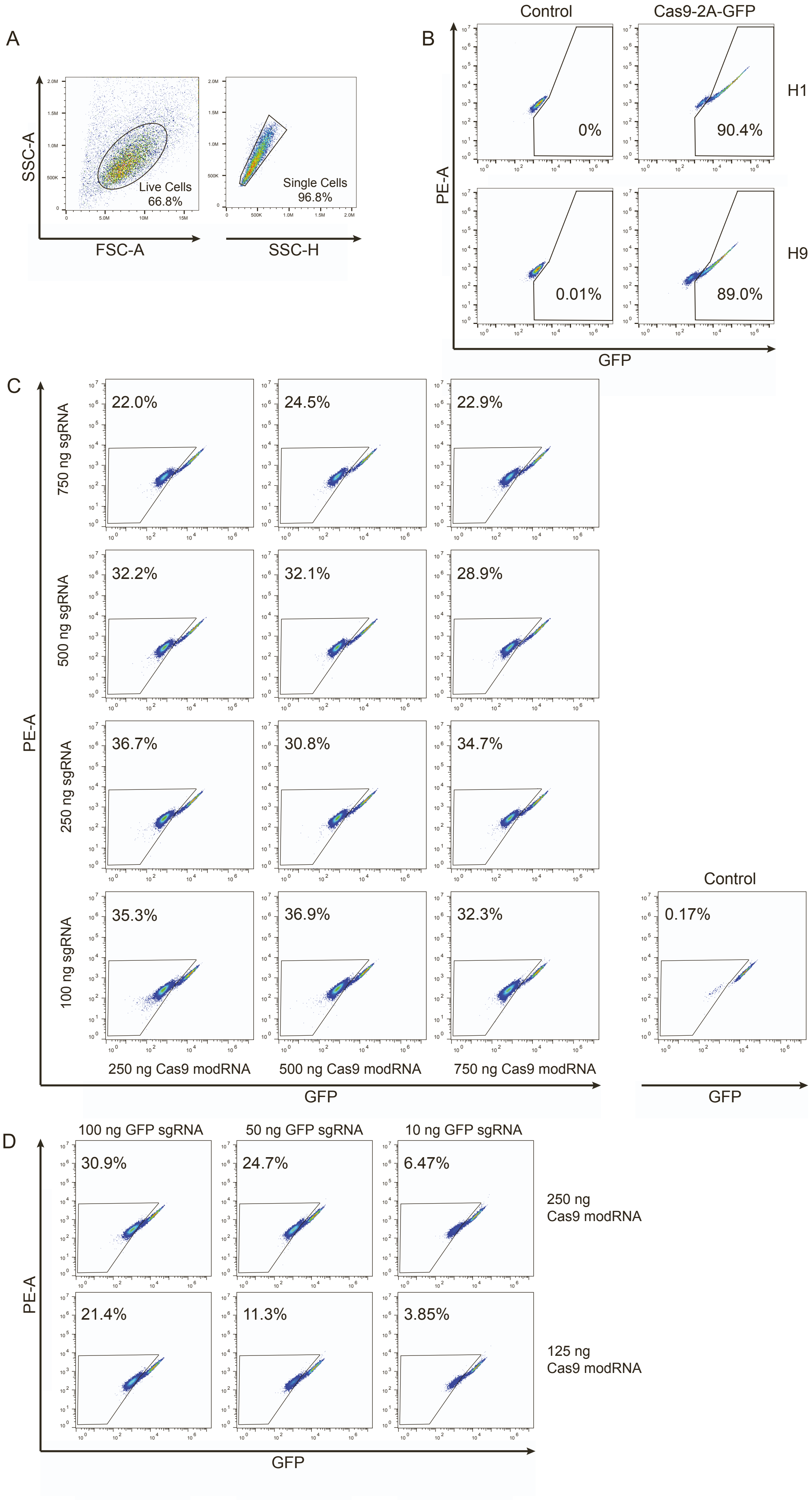
A
SSC-A
FSC-A
Live Cells
66.8%
SSC-H
Single Cells
96.8%
B
Control
Cas9-2A-GFP
0%
90.4%
H1
0.01%
89.0%
H9
PE-A
GFP
C
750 ng sgRNA
22.0%
24.5%
22.9%
500 ng sgRNA
32.2%
32.1%
28.9%
250 ng sgRNA
36.7%
30.8%
34.7%
100 ng sgRNA
35.3%
36.9%
32.3%
Control
0.17%
250 ng Cas9 modRNA
500 ng Cas9 modRNA
750 ng Cas9 modRNA
PE-A
GFP
GFP
D
100 ng GFP sgRNA
50 ng GFP sgRNA
10 ng GFP sgRNA
30.9%
24.7%
6.47%
250 ng
Cas9 modRNA
21.4%
11.3%
3.85%
125 ng
Cas9 modRNA
PE-A
GFP

Fig S1 Optimization of modRNA delivery for CRISPR mediated gene editing in hPSCs, Related to Fig. 1.

(A) Representative gating strategy used for excluding dead cells and subsequent doublet discrimination for excluding high fluorescence events during flow cytometry analysis

(B) H1 and H9 cells were cultured on iMatrix-511 in mTeSR1 using a 24-well plate and transfected with Cas9GFP modRNA using Lipofectamine Stem Transfection Reagent (1:2 ratio). 24 hours later GFP expression was analyzed by flow cytometry.

(C and D) H1 OCT4-GFP cells were cultured on iMatrix-511 in mTeSR1 and transfected with different combinations of Cas9 modRNA and GFP sgRNA. On day 4, cells were collected and GFP expression was analyzed via flow cytometry.

(C) Flow cytometry plots for combinations of 750 ng, 500 ng, and 250 ng Cas9 modRNA with either 750 ng, 500 ng, 250 ng, or 100 ng GFP sgRNA.

(D) Flow cytometry plots for combinations of 250 ng and 125 ng Cas9 modRNA with either 100 ng, 50 ng, or 10 ng GFP sgRNA. Experiment was repeated three times and representative data were shown.

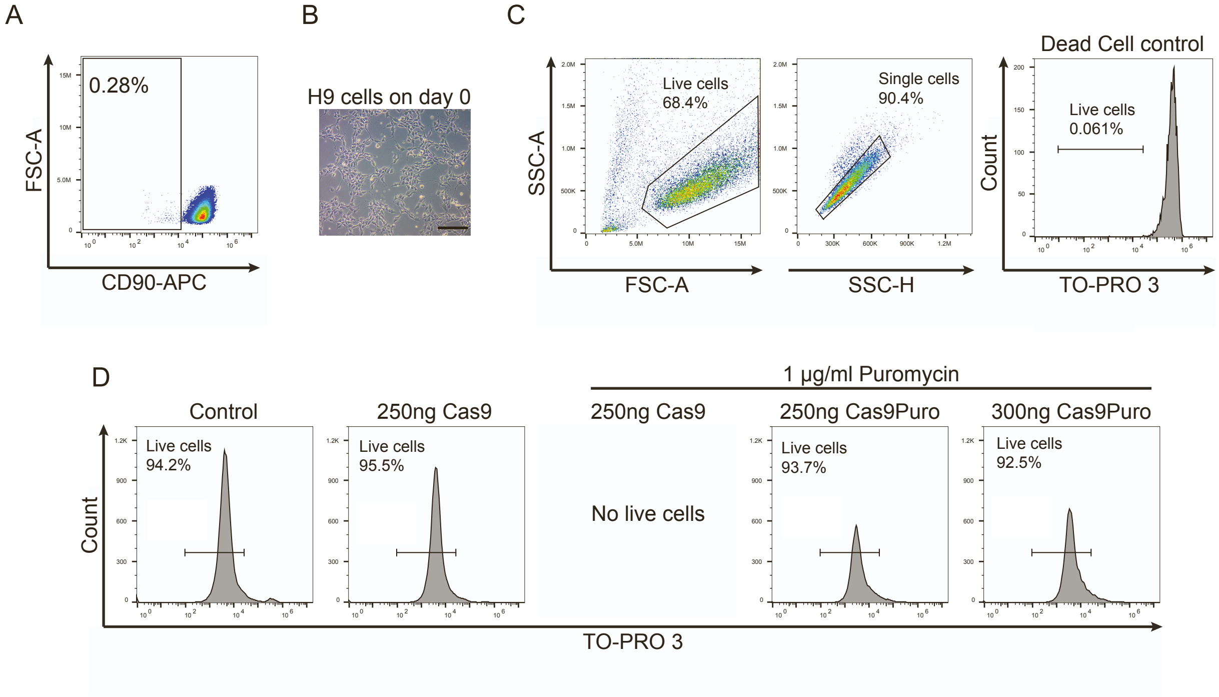
A
0.28%
FSC-A
CD90-APC
B
H9 cells on day 0
C
Live cells
68.4%
SSC-A
FSC-A
Single cells
90.4%
SSC-H
Dead Cell control
Live cells
0.061%
Count
TO-PRO 3
D
1 µg/ml Puromycin
Control
Live cells
94.2%
250ng Cas9
Live cells
95.5%
250ng Cas9
No live cells
250ng Cas9Puro
Live cells
93.7%
300ng Cas9Puro
Live cells
92.5%
Count
TO-PRO 3

Fig S2 Delivery of Cas9Puro modRNA in hPSCs, Related to Fig 2.

(A) CD90 expression in untransfected H9 cells.

(B) Representative image of H9 cells on D0 prior to transfection with Cas9Puro modRNA (scale bar = 200 μm).

(C) Representative gating strategy for counting live cells after staining with TO-PRO 3 cell viability reagent.

(D) Representative flow cytometry plots for data summarized in Fig. 2D.

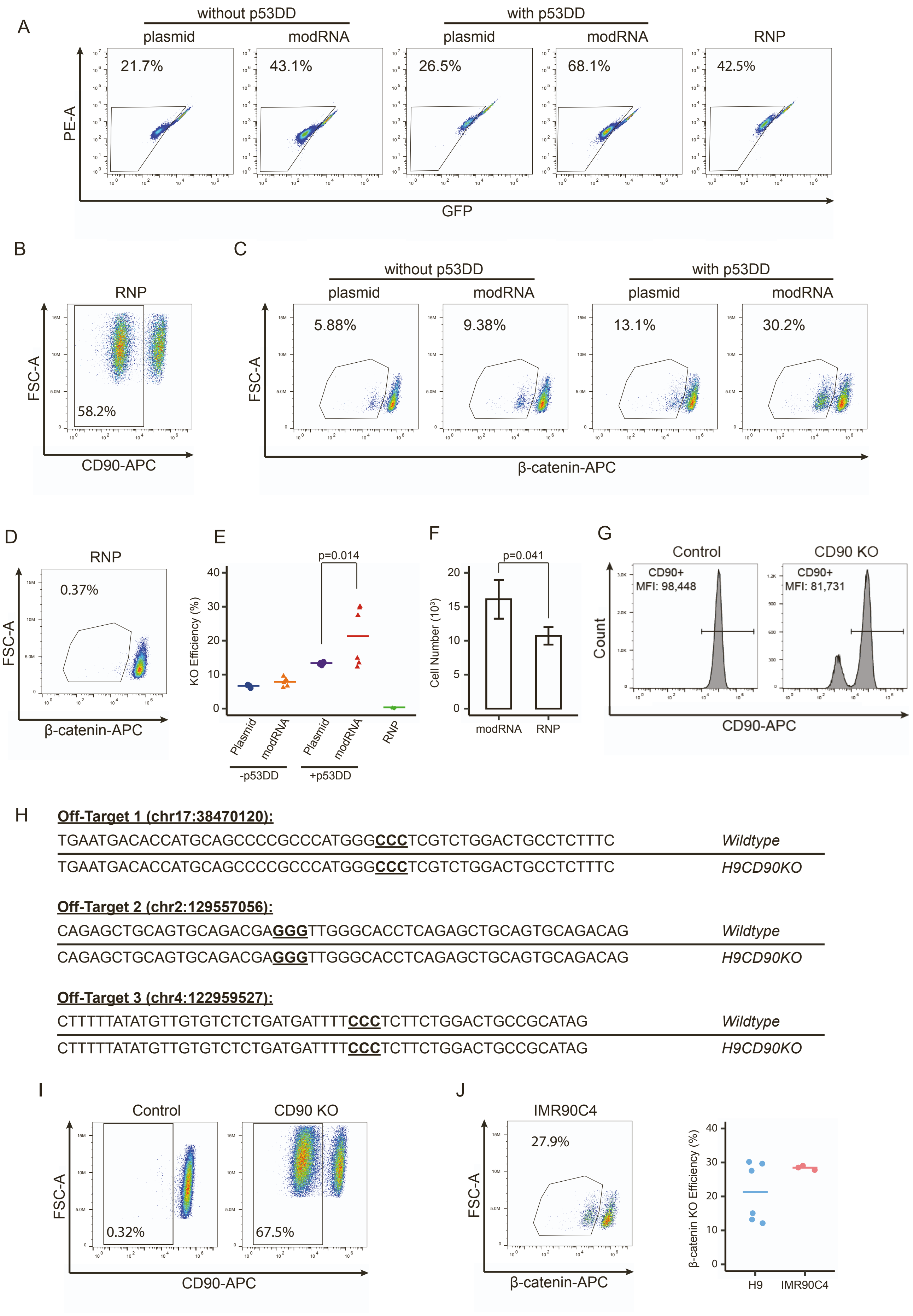
A
without p53DD
with p53DD
plasmid
modRNA
plasmid
modRNA
RNP
21.7%
43.1%
26.5%
68.1%
42.5%
PE-A
GFP
B
RNP
58.2%
FSC-A
CD90-APC
C
without p53DD
with p53DD
plasmid
modRNA
plasmid
modRNA
5.88%
9.38%
13.1%
30.2%
FSC-A
β-catenin-APC
D
RNP
0.37%
FSC-A
β-catenin-APC
E
p=0.014
KO Efficiency (%)
Plasmid
modRNA
Plasmid
modRNA
RNP
-p53DD
+p53DD
F
p=0.041
Cell Number (10³)
modRNA
RNP
G
Control
CD90 KO
CD90+
MFI: 98,448
CD90+
MFI: 81,731
Count
CD90-APC
H
Off-Target 1 (chr17:38470120):
TGAATGACACCATGCAGCCCCGCCCATGGGCCCTCGTCTGGACTGCCTCTTTC Wildtype
TGAATGACACCATGCAGCCCCGCCCATGGGCCCTCGTCTGGACTGCCTCTTTC H9CD90KO
Off-Target 2 (chr2:129557056):
CAGAGCTGCAGTGCAGACGAGGGTTGGGCACCTCAGAGCTGCAGTGCAGACAG Wildtype
CAGAGCTGCAGTGCAGACGAGGGTTGGGCACCTCAGAGCTGCAGTGCAGACAG H9CD90KO
Off-Target 3 (chr4:122959527):
CTTTTTATATGTTGTGTCTCTGATGATTTTCCCTCTTCTGGACTGCCGCATAG Wildtype
CTTTTTATATGTTGTGTCTCTGATGATTTTCCCTCTTCTGGACTGCCGCATAG H9CD90KO
I
Control
CD90 KO
0.32%
67.5%
FSC-A
CD90-APC
J
IMR90C4
27.9%
FSC-A
β-catenin-APC
β-catenin KO Efficiency (%)
H9
IMR90C4

Fig S3 Cas9 mediated gene KO with p53DD in hPSCs, Related to Fig 3.

(A) Representative flow cytometry plots of GFP KO in H1 OCT4-GFP cells transiently transfected with plasmid DNA or modRNA with or without p53DD as well as RNP method.

(B) Representative flow cytometry plot of RNP mediated CD90 KO in H9 cells on day 5 post-transfection.

(C) Representative flow plots of β-catenin KO in H9 cells transiently transfected with either plasmid DNA of modRNA with or without p53DD. Cells were collected on day 5 post-transfection and β-catenin expression was analyzed via flow cytometry.

(D) Representative flow cytometry plot of RNP mediated β-catenin KO in H9 cells on day 5 post-transfection.

(E) Aggregated β-catenin KO efficiencies across multiple replicates and batches in H9 cells, comparing results between transient transfection of plasmid DNA and modRNA-based delivery with or without p53DD as well as RNP lipofection (Plasmid: n=6; modRNA: n=6; plasmid+p53DD: n=6; modRNA+p53DD: n=6; RNP: n=6; one-way ANOVA with post-hoc Tukey's test).

(F) H9 cells were cultured in iMatrix-511 with mTeSR1 and transfected with either Cas9 modRNA and CD90 sgRNA or Cas9 protein and CD90 sgRNA. On day 2, cells were collected and stained with TO-PRO 3 cell viability reagent before being counted using a flow cytometer (n=3; unpaired student's T-test).

(G) Flow cytometry analyses of CD90 expression in untransfected and transfected H9 cells with Cas9 modRNA and CD90 sgRNA.

(H) Off-target analysis of CD90 KO H9 cells generated using CRISPR modRNA cocktail with p53DD.

(I) Representative flow cytometry plot of CD90 KO in IMR90C4 cells using CRISPR modRNA cocktail with p53DD.

(J) IMR90C4 cells cultured on iMatrix-511 in mTeSR1 were transfected with Cas9Puro modRNA, CTNNB1 sgRNA, and p53DD modRNA. On day 5, cells were collected, and β-catenin expression was analyzed via flow cytometry. Representative flow cytometry plot and quantification (H9: n=6; IMR90C4: n=3).

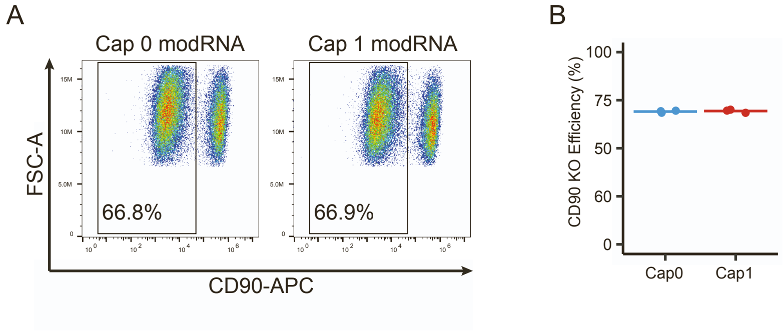
A
Cap 0 modRNA
Cap 1 modRNA
FSC-A
66.8%
66.9%
CD90-APC
B
CD90 KO Efficiency (%)
100
75
50
60
0
Cap0
Cap1

Fig S4 Both cap 1 and cap 0 modRNA structures can mediate efficient genome editing in hPSCs, Related to Fig 3.

IMR90C4 cells were cultured on iMatrix-511 in mTeSR1 using a 12-well plate and transfected with 600 ng Cas9Puro modRNA (Cap0 or Cap1), 200 ng CD90 sgRNA, and 200 ng p53DD modRNA. On day 5, cells were collected and CD90 expression was analyzed by flow cytometry.

(A) Representative flow cytometry plot.

(B) Quantification of flow cytometry results from day 5 cells (n=3).

Table S1: Oligonucleotides used in this paper for gene cloning, sequencing editing sites, and gRNA sequences.

**Gene Cloning into modRNAc0 vector**	
Cas9	Forward: CATGGCATGCGAATTCATGGACAAGAAGTACTCCATTGGGC<br>Reverse: AAGCGAGCTCACTAGTTTAGTCTCCACCGAGCTGAGAG
Cas9-2A-Puro	Forward: CATGGCATGCGAATTCGCCACCATGGATTACAAAGACG<br>Reverse: AAGCGAGCTCACTAGTTCAGGCACCGGGCTTGCG
Cas9-2A-GFP	Forward: CATGGCATGCGAATTCATGGACAAGAAGTACTCCATTGGGC<br>Reverse: AAGCGAGCTCACTAGTTTACTTGTACAGCTCGTCCATGCC
p53DD	Forward: CATGGCATGCGAATTCGCCACCATGACTGCCATGG<br>Reverse: AAGCGAGCTCACTAGTTCAGTCTGAGTCAGGCCCC
ABE8e-GFP	Forward: CATGGCATGCGAATTCGCCACCATGAAACGGACAGC<br>Reverse: AAGCGAGCTCACTAGTTTATACCTTACGCTTCTTCTTTGGC
**Sequencing primers for editing sites**	
CD90 On-target	Forward: ATCTCTCCACTTCAGGTGGGT<br>Reverse: TGTATTTGCTGGTGAAGTTGGT
CD90 Off-target 1	Forward: AGAGAGGGTGTCAGGGAGGT<br>Reverse: CTAAAAAGCCGCGAAGACAG
CD90 Off-target 2	Forward: CTCACAGGCATTCACAAGGA<br>Reverse: GCAGGAGTCACTGTCTGCAC
CD90 Off-target 3	Forward: TTGTGGACCTGCATGTTTGT<br>Reverse: CACAAACACTACAGAGGTTTTGTATTC
B2M splice donor site	Forward: GCGTTTAATATAAGTGGAGGCG<br>Reverse: CACCAAGGAGAACTTGGAGAAG
**gRNA sequences**	
GFP sgRNA	GGGCGAGGAGCTGTTCACCG
CD90 sgRNA-1	CATGGCGGCAGTCCAGACGA
CD90 sgRNA-2	GCCTTCACTAGCAAGGACGA
B2M sgRNA	ACTCACGCTGGATAGCCTCC
β-catenin sgRNA	GAAACAGCTCGTTGTACCGC

Data S1: Full sequence and map of plasmid XLoneV3-ABE8e. Related to the STAR Methods.

Full sequence (TRE3G promoter, ABE8e, 2A, EGFP; Tet-On 3G, EF1a core promoter**):**
atcacctcgagtttactccctatcagtgatagagaacgtatgaagagtttactccctatcagtgatagagaacgtatgcagactttactccctatcagtgatagagaacgtata
aggagtttactccctatcagtgatagagaacgtatgaccagtttactccctatcagtgatagagaacgtatctacagtttactccctatcagtgatagagaacgtatatccagtt
tactccctatcagtgatagagaacgtataagctttgcttatgtaaaccagggcgcctataaaagagtgctgatttttgagtaaacttcaattccacaacacttttgtcttatacca
actttccgtaccacttcctaccctcgtaaaggtaccgccaccatgaaacggacagccgacggaagcgagttcgagtcaccaaagaagaagcggaaagtctctgaggtg
gagttttcccacgagtactggatgagacatgccctgaccctggccaagagggcacgggatgagagggaggtgcctgtgggagccgtgctggtgctgaacaatagagt
gatcggcgagggctggaacagagccatcggcctgcacgacccaacagcccatgccgaaattatggccctgagacagggcggcctggtcatgcagaactacagactg
attgacgccaccctgtacgtgacattcgagccttgcgtgatgtgcgccggcgccatgatccactctaggatcggccgcgtggtgtttggatggagaaattctaaaagagg
cgccgcaggctccctgatgaacgtgctgaactaccccggcatgaatcaccgcgtcgaaattaccgagggaatcctggcagatgaatgtgccgccctgctgtgcgatttct
atcggatgcctagacaggtgttcaatgctcagaagaaggcccagagctccatcaactccggaggatctagcggaggctcctctggctctgagacacctggcacaagcg
agagcgcaacacctgaaagcagcgggggcagcagcgggggtcagacaagaagtacagcatcggcctggccatcggcaccaactctgtgggctgggccgtgatca
ccgacgagtacaaggtgcccagcaagaaattcaaggtgctgggcaacaccgaccggcacagcatcaagaagaacctgatcggagccctgctgttcgacagcggcga
aacagccgaggccacccggctgaagagaaccgccagaagaagatacaccagacggaagaaccggatctgctatctgcaagagatcttcagcaacgagatggccaa
ggtggacgacagcttcttccacagactggaagagtccttcctggtggaagaggataagaagcacgagcggcaccccatcttcggcaacatcgtggacgaggtggccta
ccacgagaagtaccccaccatctaccacctgagaaagaaactggtggacagcaccgacaaggccgacctgcggctgatctatctggccctggcccacatgatcaagtt
ccggggccacttcctgatcgagggcgacctgaaccccgacaacagcgacgtggacaagctgttcatccagctggtgcagacctacaaccagctgttcgaggaaaacc
ccatcaacgccagcggcgtggacgccaaggccatcctgtctgccagactgagcaagagcagacggctggaaaatctgatcgcccagctgcccggcgagaagaaga
atggcctgttcggaaacctgattgccctgagcctgggcctgacccccaacttcaagagcaacttcgacctggccgaggatgccaaactgcagctgagcaaggacaccta
cgacgacgacctggacaacctgctggcccagatcggcgaccagtacgccgacctgtttctggccgccaagaacctgtccgacgccatcctgctgagcgacatcctgag
agtgaacaccgagatcaccaaggcccccctgagcgcctctatgatcaagagatacgacgagcaccaccaggacctgaccctgctgaaagctctcgtgcggcagcagc
tgcctgagaagtacaaagagattttcttcgaccagagcaagaacggctacgccggctacattgacggcggagccagccaggaagagttctacaagttcatcaagcccat

cctggaaaagatggacggcaccgaggaactgctcgtgaagctgaacagagaggacctgctgcggaagcagcggaccttcgacaacggcagcatcccccaccagat
ccacctgggagagctgcacgccattctgcggcggcaggaagatttttacccattcctgaaggacaaccgggaaaagatcgagaagatcctgaccttccgcatcccctac
tacgtgggccctctggccaggggaaacagcagattcgcctggatgaccagaaagagcgaggaaaccatcacccccctggaacttcgaggaagtggtggacaagggcg
cttccgcccagagcttcatcgagcggatgaccaacttcgataagaacctgcccaacgagaaggtgctgcccaagcacagcctgctgtacgagtacttcaccgtgtataac
gagctgaccaaagtgaaatacgtgaccgagggaatgagaaagcccgccttcctgagcggcgagcagaaaaaggccatcgtggacctgctgttcaagaccaaccgga
aagtgaccgtgaagcagctgaaagaggactacttcaagaaaatcgagtgcttcgactccgtggaaatctccggcgtggaagatcggttcaacgcctccctgggcacata
ccacgatctgctgaaaattatcaaggacaaggacttcctggacaatgaggaaaacgaggacattctggaagatatcgtgctgaccctgacactgtttgaggacagagaga
tgatcgaggaacggctgaaaacctatgcccacctgttcgacgacaaagtgatgaagcagctgaagcggcggagatacaccggctggggcaggctgagccggaagct
gatcaacggcatccgggacaagcagtccggcaagacaatcctggatttcctgaagtccgacggcttcgccaacagaaacttcatgcagctgatccacgacgacagcct
gacctttaaagaggacatccagaaagcccaggtgtccggccagggcgatagcctgcacgagcacattgccaatctggccggcagccccgccattaagaagggcatcc
tgcagacagtgaaggtggtggacgagctcgtgaaagtgatgggccggcacaagcccgagaacatcgtgatcgaaatggccagagagaaccagaccacccagaagg
gacagaagaacagccgcgagagaatgaagcggatcgaagagggcatcaaagagctgggcagccagatcctgaaagaacaccccgtggaaaacacccagctgcag
aacgagaagctgtacctgtactacctgcagaatgggcgggatatgtacgtggaccaggaactggacatcaaccggctgtccgactacgatgtggaccatatcgtgcctc
agagctttctgaaggacgactccatcgacaacaaggtgctgaccagaagcgacaagaaccggggcaagagcgacaacgtgccctccgaagaggtcgtgaagaagat
gaagaactactggcggcagctgctgaacgccaagctgattacccagagaaagttcgacaatctgaccaaggccgagagaggcggcctgagcgaactggataaggcc
ggcttcatcaagagacagctggtggaaacccggcagatcacaaagcacgtggcacagatcctggactcccggatgaacactaagtacgacgagaatgacaagctgat
ccgggaagtgaaagtgatcaccctgaagtccaagctggtgtccgatttccggaaggatttccagttttacaaagtgcgcgagatcaacaactaccaccacgcccacgac
gcctacctgaacgccgtcgtgggaaccgccctgatcaaaaagtaccctaagctggaaagcgagttcgtgtacggcgactacaaggtgtacgacgtgcggaagatgatc
gccaagagcgagcaggaaatcggcaaggctaccgccaagtacttcttctacagcaacatcatgaactttttcaagaccgagattaccctggccaacggcgagatccgga
agcggcctctgatcgagacaaacggcgaaaccggggagatcgtgtgggataagggccgggattttgccaccgtgcggaaagtgctgagcatgccccaagtgaatatc
gtgaaaaagaccgaggtgcagacaggcggcttcagcaaagagtctatcctgcccaagaggaacagcgataagctgatcgccagaaagaaggactgggaccctaaga
agtacggcggcttcgacagccccaccgtggcctattctgtgctggtggtggccaaagtggaaaagggcaagtccaagaaactgaagagtgtgaaagagctgctgggg
atcaccatcatggaaagaagcagcttcgagaagaatcccatcgactttctggaagccaagggctacaaagaagtgaaaaaggacctgatcatcaagctgcctaagtact
ccctgttcgagctggaaaacggccggaagagaatgctggcctctgccggcgaactgcagaagggaaacgaactggccctgccctccaaatatgtgaacttcctgtacct
ggccagccactatgagaagctgaagggctcccccgaggataatgagcagaaacagctgtttgtggaacagcacaagcactacctggacgagatcatcgagcagatca
gcgagttctccaagagagtgatcctggccgacgctaatctggacaaagtgctgtccgcctacaacaagcaccgggataagcccatcagagagcaggccgagaatatca
tccacctgtttaccctgaccaatctgggagcccctgccgccttcaagtactttgacaccaccatcgaccggaagaggtacaccagcaccaaagaggtgctggacgccac
cctgatccaccagagcatcaccggcctgtacgagacacggatcgacctgtctcagctgggaggtgactctggcggctcaaaaagaaccgccgacggcagcgaattcg
agcccaagaagaagaggaaagtcgctagcggcagcggcgccactaacttctccctgttgaaacaagcaggggatgtcgaagagaatcccgggccaatggtgagcaa
gggcgaggagctgttcaccggggtggtgcccatcctggtcgagctggacggcgacgtaaacggccacaagttcagcgtgtccggcgagggcgagggcgatgccac
ctacggcaagctgaccctgaagttcatctgcaccaccggcaagctgcccgtgccctggcccaccctcgtgaccaccctgacctacggcgtgcagtgcttcagccgctac
cccgaccacatgaagcagcacgacttcttcaagtccgccatgcccgaaggctacgtccaggagcgcaccatcttcttcaaggacgacggcaactacaagacccgcgcc
gaggtgaagttcgagggcgacaccctggtgaaccgcatcgagctgaagggcatcgacttcaaggaggacggcaacatcctggggcacaagctggagtacaactaca
acagccacaacgtctatatcatggccgacaagcagaagaacggcatcaaggtgaacttcaagatccgccacaacatcgaggacggcagcgtgcagctcgccgaccac
taccagcagaacacccccatcggcgacggccccgtgctgctgcccgacaaccactacctgagcacccagtccgccctgagcaaagaccccaacgagaagcgcgatc
acatggtcctgctggagttcgtgaccgccgccgggatcactctcggcatggacgagctgtacaagccaaagaagaagcgtaaggtataagtacaagtaagaattccgat
catattcaataacccactagtagaccacctcccctgcgagctaagctggacagccaatgacgggtaagagagtgacatttttcactaacctaagacaggagggccgtcag
agctactgcctaatccaaagacgggtaaaagtgataaaaatgtatcactccaacctaagacaggcgcagcttccgagggatttgagatccagacatgataagatacattga
tgagtttggacaaaccaaaactagaatgcagtgaaaaaaatgccttatttgtgaaatttgtgatgctattgccttatttgtaaccattataagctgcaataaacaagtttgatatct
ataacaagaaaatatatatataataagttatcacgtaagtagaacatgaaataacaatataattatcgtatgagttaaatcttaaaagtcacgtaaaagataatcatgcgtcatttt
gactcacgcggtcgttatagttcaaaatcagtgacacttaccgcattgacaagcacgcctcacgggagctccaagcggcgactgagatgtcctaaatgcacagcgacgg
attcgcgctatttagaaagagagagcaatatttcaagaatgcatgcgtcaattttacgcagactatctttctagggttaagaattcactggccgtcgttttacaacgtcgtgact
gggaaaaccctggcgttacccaacttaatcgccttgcagcacatccccctttcgccagctggcgtaatagcgaagaggcccgcaccgatcgcccttcccaacagttgcg
cagcctgaatggcgaatggcgcctgatgcggtattttctccttacgcatctgtgcggtatttcacaccgcatatggtgcactctcagtacaatctgctctgatgccgcatagtt
aagccagccccgacacccgccaacacccgctgacgcgccctgacgggcttgtctgctcccggcatccgcttacagacaagctgtgaccgtctccgggagctgcatgtg
tcagaggttttcaccgtcatcaccgaaacgcgcgagacgaaagggcctcgtgatacgcctatttttataggttaatgtcatgataataatggtttcttagacgtcaggtggca
cttttcggggaaatgtgcgcggaacccctatttgtttatttttctaaatacattcaaatatgtatccgctcatgagacaataaccctgataaatgcttcaataatattgaaaaagga
agagtatgagtattcaacatttccgtgtcgcccttattcccttttttgcggcattttgccttcctgtttttgctcacccagaaacgctggtgaaagtaaaagatgctgaagatcagt
tgggtgcacgagtgggttacatcgaactggatctcaacagcggtaagatccttgagagttttcgccccgaagaacgttttccaatgatgagcacttttaaagttctgctatgt
ggcgcggtattatcccgtattgacgccgggcaagagcaactcggtcgccgcatacactattctcagaatgacttggttgagtactcaccagtcacagaaaagcatcttacg
gatggcatgacagtaagagaattatgcagtgctgccataaccatgagtgataacactgcggccaacttacttctgacaacgatcggaggaccgaaggagctaaccgcttt
tttgcacaacatgggggatcatgtaactcgccttgatcgttgggaaccggagctgaatgaagccataccaaacgacgagcgtgacaccacgatgcctgtagcaatggca
acaacgttgcgcaaactattaactggcgaactacttactctagcttcccggcaacaattaatagactggatggaggcggataaagttgcaggaccacttctgcgctcggcc
cttccggctggctggtttattgctgataaatctggagccggtgagcgtgggtctcgcggtatcattgcagcactggggccagatggtaagccctcccgtatcgtagttatct
acacgacggggagtcaggcaactatggatgaacgaaatagacagatcgctgagataggtgcctcactgattaagcattggtaactgtcagaccaagtttactcatatatac
tttagattgatttaaaacttcatttttaatttaaaaggatctaggtgaagatcctttttgataatctcatgaccaaaatcccttaacgtgagttttcgttccactgagcgtcagacccc

gtagaaaagatcaaaggatcttcttgagatcctttttttctgcgcgtaatctgctgcttgcaaacaaaaaaaccaccgctaccagcggtggtttgtttgccggatcaagagcta
ccaactctttttccgaaggtaactggcttcagcagagcgcagataccaaatactgttcttctagtgtagccgtagttaggccaccacttcaagaactctgtagcaccgcctac
atacctcgctctgctaatcctgttaccagtggctgctgccagtggcgataagtcgtgtcttaccgggttggactcaagacgatagttaccggataaggcgcagcggtcggg
ctgaacggggggttcgtgcacacagcccagcttggagcgaacgacctacaccgaactgagatacctacagcgtgagctatgagaaagcgccacgcttcccgaaggga
gaaaggcggacaggtatccggtaagcggcagggtcggaacaggagagcgcacgagggagcttccagggggaaacgcctggtatctttatagtcctgtcgggtttcgc
cacctctgacttgagcgtcgatttttgtgatgctcgtcaggggggcggagcctatggaaaaacgccagcaacgcggcctttttacggttcctggccttttgctggccttttgc
tcacatgttctttcctgcgttatcccctgattctgtggataaccgtattaccgcctttgagtgagctgataccgctcgccgcagccgaacgaccgagcgcagcgagtcagtg
agcgaggaagcggaagagcgcccaatacgcaaaccgcctctccccgcgcgttggccgattcattaatgcagctggcacgacaggtttcccgactggaaagcgggca
gtgagcgcaacgcaattaatgtgagttagctcactcattaggcaccccaggctttacactttatgcttccggctcgtatgttgtgtggaattgtgagcggataacaatttcaca
caggaaacagctatgaccatgattacgccaaggtcgacttaaccctagaaagataatcatattgtgacgtacgttaaagataatcatgcgtaaaattgacgcatgtgttttatc
ggtctgtatatcgaggtttatttattaatttgaatagatattaagttttattatatttacacttacatactaataataaattcaacaaacaatttatttatgtttatttatttattaaaaaaaaa
caaaaactcaaaatttcttctataaagtaacaaaacttttagcagtgaaaaaaatgctttatttgtgaaatttgtgatgctattgctttatttgtaaccattataagctgcaataaaca
agttaactaaacgggccctctagatcaggcaccgggcttgcgggtcatgcaccaggtgcgcggtccttcgggcacctcgacgtcggcggtgacggtgaagccgagcc
gctcgtagaaggggaggttgcggggcgcggaggtctccaggaaggcgggcaccccggcgcgctcggccgcctccactccggggagcacgacggcgctgcccag
acccttgccctggtggtcgggcgagacgccgacggtggccaggaaccacgcgggctccttgggccggtgcggcgccaggaggccttccatctgttgctgcgcggcc
agccgggaaccgctcaactcggccatgcgcgggccgatctcggcgaacaccgcccccgcttcgacgctctccggcgtggtccagaccgccaccgcggcgccgtcgt
ccgcgacccacaccttgccgatgtcgagcccgacgcgcgtgaggaagagttcttgcagctcggtgacccgctcgatgtggcggtccggatcgacggtgtggcgcgtg
gcggggtagtcggcgaacgcggcggcgagggtgcgtacggccctggggacgtcgtcgcgggtggcgaggcgcaccgtgggcttgtactcggtcatctcgagccta
gggccgggattctcctccacgtcaccgcatgttagaagacttcctctgccctctccgctgcccccggggagcatgtcaaggtcaaaatcgtcaagagcgtcagcaggca
gcatatcaaggtcaaagtcgtcaagggcatcggctgggagcatgtctaagtcaaaatcgtcaagggcgtcggtcggcccgccgctttcgcactttagctgtttctccaggc
cacatatgattagttccaggccgaaaaggaaggcaggttcggctccctgccggtcgaacagctcaattgcttgtttcagaagtgggggcatagaatcggtggtaggtgtct
ctctttcctcttttgctacttgatgctcctgttcctccaatacgcagcccagtgtaaagtggcccacggcggacagagcgtacagtgcgttctccagggagaagccttgctga
cacaggaacgcgagctgattttccagggtttcgtactgtttctctgttgggcgggtgccgagatgcactttagccccgtcgcgatgtgagaggagagcacagcggtatga
cttggcgttgttccgcagaaagtcttgccatgactcgccttccagggggcaggagtgggtatgatgcctgtccagcatctcgattggcagggcatcgagcagggcccgct
tgttcttcacgtgccagtacagggtaggctgctcaactcccagcttttgagcgagtttccttgtcgtcaggccttcgataccgactccattgagtaattccagagcagagtttat
gactttgctcttgtccagtctagacatcttatcgtcatcgtctttgtaatccatggtggcggatcccgcgtcacgacacctgtgttctggcggcaaacccgttgcgaaaaaga
acgttcacggcgactactgcacttatatacggttctcccccaccctcgggaaaaaggcggagccagtacacgacatcactttcccagtttaccccgcgccaccttctctag
gcaccggttcaattgccgacccctccccccaacttctcggggactgtgggcgatgtgcgctctgcccactgacgggcaccggagccactcgagtggaatt

Map:

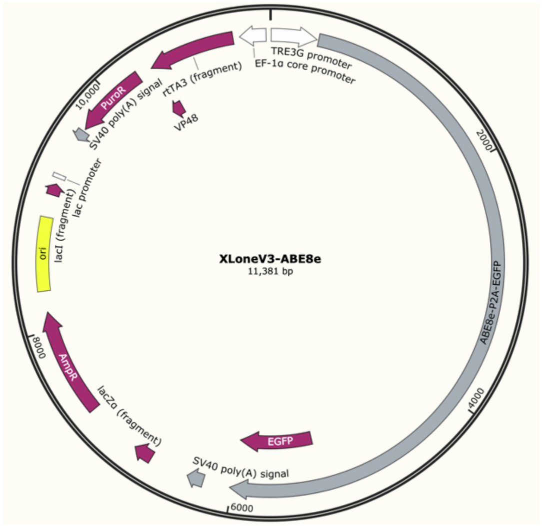

Supplement: Documment S1. Figures S1–S3, Table S1, and Data S1 [file mmc1.pdf]
