# Supplementary material for: Robust genome editing via modRNA-based Cas9 or base editor in human pluripotent stem cells
Source: Cell Rep Methods. 2022 Sep 7;2(9):100290. doi: 10.1016/j.crmeth.2022.100290 (PMC9499999; doi:10.1016/j.crmeth.2022.100290)

Cell Reports Methods

Article

# Robust genome editing via modRNA-based Cas9 or base editor in human pluripotent stem cells

## Graphical abstract

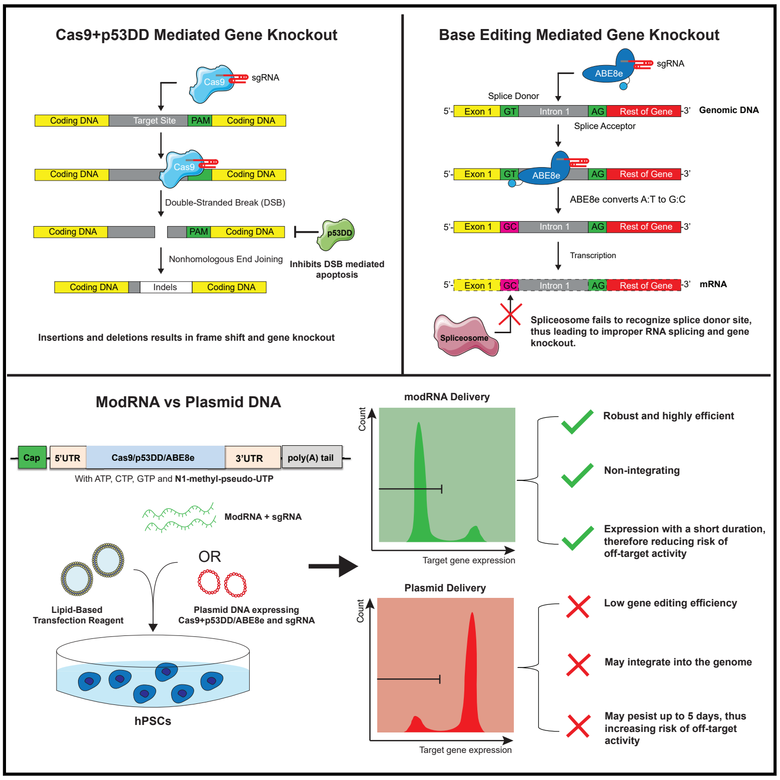


## Highlights

- Transfection of hPSCs with Cas9 modRNA yielded 90% transfection efficiency
- Gene editing of hPSCs via Cas9/p53DD modRNA yielded up to 84% knockout efficiency
- Cas9/p53DD modRNA method was better than plasmid- and RNP-based methods
- ABE8e modRNA achieved 5-fold knockout efficiency relative to the plasmid ABE8e

## Authors

Tahir Haideri, Alessandro Howells, Yuqian Jiang, Jian Yang, Xiaoping Bao, Xiaojun Lance Lian

## Correspondence

bao61@purdue.edu (X.B.),
Lian@psu.edu (X.L.L.)

## In brief

Haideri et al. develop non-integrating modRNA-based CRISPR systems for achieving robust and efficient gene knockouts in hPSCs. They further establish an ABE8e base editor modRNA protocol to disrupt the splice donor site. These non-integrating approaches can preserve genome integrity and significantly enhance knockout efficiency.

Haideri et al., 2022, Cell Reports Methods *2*, 100290
September 19, 2022 © 2022 The Author(s).
https://doi.org/10.1016/j.crmeth.2022.100290

CellPress

Cell Reports Methods

CellPress
OPEN ACCESS

Article

# Robust genome editing via modRNA-based Cas9 or base editor in human pluripotent stem cells

Tahir Haideri,[1,5] Alessandro Howells,[1,5] Yuqian Jiang,[1] Jian Yang,[1,3] Xiaoping Bao,[4,*] and Xiaojun Lance Lian[1,2,3,6,*]
[1]Department of Biomedical Engineering, Pennsylvania State University, University Park, PA 16802, USA
[2]Department of Biology, Pennsylvania State University, University Park, PA 16802, USA
[3]The Huck Institutes of the Life Sciences, Pennsylvania State University, University Park, PA 16802, USA
[4]Davidson School of Chemical Engineering, Purdue University, West Lafayette, IN 47907, USA
[5]These authors contributed equally
[6]Lead contact
*Correspondence: bao61@purdue.edu (X.B.), Lian@psu.edu (X.L.L.)
https://doi.org/10.1016/j.crmeth.2022.100290

**MOTIVATION** Robust and efficient methods for knocking out genes in stem cells are indispensable in understanding the function of a gene during stem cell differentiation. Plasmid-based CRISPR systems can be used to generate gene knockouts, but the efficiency is low, and plasmid DNA may integrate into the genome and thus compromise genome integrity. We sought to develop non-integrating and efficient modified mRNA (modRNA)-based CRISPR systems (Cas9 or base editor) that can be used to achieve robust gene knockouts in both human embryonic stem cells and induced pluripotent stem cells to circumvent the challenges faced by plasmid-based CRISPR systems.

## SUMMARY

CRISPR systems have revolutionized biomedical research because they offer an unprecedented opportunity for genome editing. However, a bottleneck of applying CRISPR systems in human pluripotent stem cells (hPSCs) is how to deliver CRISPR effectors easily and efficiently. Here, we developed modified mRNA (modRNA)-based CRIPSR systems that utilized Cas9 and p53DD or a base editor (ABE8e) modRNA for the purposes of knocking out genes in hPSCs via simple lipid-based transfection. ABE8e modRNA was employed to disrupt the splice donor site, resulting in defective splicing of the target transcript and ultimately leading to gene knockout. Using our modRNA CRISPR systems, we achieved 73.3% ± 11.2% and 69.6 ± 3.8% knockout efficiency with Cas9 plus p53DD modRNA and ABE8e modRNA, respectively, which was significantly higher than the plasmid-based systems. In summary, we demonstrate that our non-integrating modRNA-based CRISPR methods hold great promise as more efficient and accessible techniques for genome editing of hPSCs.

## INTRODUCTION

CRISPR-Cas systems are used for genome editing in a wide variety of cell types and are useful for high-throughput genome-wide screens (Xu et al., 2020; Yilmaz et al., 2018). Cas9 is the most-used endonuclease of the CRISPR-Cas family (Cong et al., 2013; Jinek et al., 2012; Mali et al., 2013) and can precisely cleave genomic DNA via double-stranded breaks (DSBs) when paired with a programmable single guide RNA (sgRNA) with minimal off-target effects. Repair of DSBs can occur through one of the two intrinsic pathways in mammalian cells: non-homologous end joining (NHEJ) and homology-directed repair (HDR). NHEJ results in insertions or deletions (indels), which can lead to frameshift mutations and, consequently, gene knockout (KO) (Cong et al., 2013; Mali et al., 2013). Alternatively, co-delivery of a donor DNA template can precisely introduce desired sequence edits via the HDR pathway. DNA cleavage is mediated by the HNH and RuvC domains of the Cas9 protein (Jinek et al., 2012; Sternberg et al., 2015). Mutations in these domains result in a catalytically inactive Cas9 (dCas9), which allows for a more general platform for RNA-guided, genomic delivery of a wide variety of covalently tethered effector proteins, among them being base editors (Komor et al., 2016). The two primary base editors used in practice are based on either adenosine or cytidine deaminases. They are also known as adenine base editors (ABEs) (Gaudelli et al., 2017, 2020; Richter et al., 2020) or cytidine


© 2022 The Author(s).
This is an open access article under the CC BY license (http://creativecommons.org/licenses/by/4.0/).

base editors (CBEs) (Koblan et al., 2018; Komor et al., 2016). ABEs specifically convert deoxyadenosine (dA) to deoxyinosine (dI), which, in turn, is repaired to deoxyguanosine (dG). CBEs, on the other hand, convert deoxycytidine (dC) to deoxyuridine (dU), which gets repaired to deoxythymidine (dT). When the adenosine or cytidine deaminase is covalently tethered to a dCas9, this enables researchers to introduce a genomic point mutation at high fidelity without DSBs, thus significantly reducing the risk of potentially detrimental indels and chromosomal rearrangements at off-target sites. ABEs and CBEs have been leveraged to correct disease-related point mutations and for gene KO purposes at relatively high efficiencies and specificities (Antoniou et al., 2021; Kluesner et al., 2021).

Human pluripotent stem cells (hPSCs) can be expanded almost indefinitely while still maintaining their ability to differentiate into all somatic cell lineages (Jiang et al., 2021; Lian et al., 2012, 2013, 2014, 2015). They can be utilized to generate *in vitro* cell culture models for studying human development and disease modeling when coupled with CRISPR-Cas9 systems (Antoniou et al., 2021). Despite their remarkable potential, the current state-of-the-art methods for delivering CRISPR components into hPSCs are far from ideal. Virus-mediated gene delivery is considered as an efficient method for the delivery of CRISPR components into most cell types (Hsu et al., 2019). Commonly used viral vectors include lentiviruses, adeno-associated viruses (AAVs), and adenoviruses. Lentiviruses are normally integrating, which can increase the risk of tumorigenicity, and therefore, hPSC lines with lentiviral integrations may be counterproductive during their use in cell-based therapies. Additionally, hPSCs were reported to be resistant to lentiviral infection due to unique intrinsic immunity (Wu et al., 2018). AAVs and adenoviruses are two non-integrating alternatives to lentiviruses. However, adenoviruses are known to trigger high levels of innate immune response in transduced cells, which can lead to inflammation. AAVs have a relatively low packaging limit (~4.7 kb), making it difficult to deliver CRISPR components. Additionally, AAVs and adenoviruses are laborious to produce and require the use of specialized equipment for their purification.

Non-viral state-of-the-art methods for delivering CRISPR components into hPSCs include a variety of physical and chemical delivery strategies. Electroporation and lipid nanoparticles (LNPs) are two commonly used non-viral delivery methods that use plasmid DNA for the delivery of CRISPR components via either nucleofection or transfection reagents (Liu et al., 2016). These methods, however, have low transfection efficiency and can be cytotoxic to cells. Ribonucleoproteins (RNPs), on the other hand, consisting of Cas9 protein complexed with a sgRNA, have also been shown to efficiently edit the genome (Martin et al., 2019). Cas9 protein is commercially available; however, CBEs and ABEs are not, and producing purified samples of these proteins can be cumbersome and not feasible for many labs.

An emerging alternative to these approaches above is the use of chemically modified RNA (modRNA) for the delivery of CRISPR effectors into cells. modRNA is coined "modified" because chemically modified nucleotides are used during *in vitro* transcription synthesis. It has been shown that when un-modified regular mRNA is introduced to mammalian cells, it is not stable, and it triggers the cellular immune response (Hadas et al., 2019). However, modRNA has increased stability and lower immunogenicity (Karikó et al., 2005, 2008). Further optimization of modRNA led to the discovery of replacing uridine with N1-methyl-pseudouridine to achieve robust translation of modRNA due to enhanced ribosomal recruitment (Svitkin et al., 2017). Additionally, the use of modRNA-based gene overexpression has been shown to directly program hPSCs to desired cell types, such as hematopoietic progenitors (Suknuntha et al., 2018). modRNA technology has also been used for gene editing. For example, researchers discovered that uridine depletion and chemical modification increased Cas9 mRNA activity and reduced immunogenicity in cell lines and primary $CD34^+$ cells (Vaidyanathan et al., 2018). Scientists also reported that uridine depleted ABE mRNA with 5-methoxyuridine mediates robust editing at various cellular genomic sites (Jiang et al., 2020), achieving higher efficiency than gene editing using regular unmodified mRNA (Sürün et al., 2020). The use of modRNA-based CRISPR systems in hPSCs, however, remained unexplored. All said, the use of modRNA to encode and deliver CRISPR systems carries several advantages over previous methods: (1) it is non-integrating; (2) it does not require transport across the nuclear membrane for expression (as is the case with plasmid delivery), therefore increasing transfection efficiency; (3) it is relatively quick and easy to perform; (4) it requires a minimal starting cell population; and (5) it is only transiently expressed, thus greatly reducing the risk of off-target activity.

In this study, we developed modRNA-based genome-editing systems for hPSCs that utilize simple lipid-based transfection of sgRNAs, with Cas9 and p53DD or ABE8e modRNA. Using our optimized protocol, we were able to achieve up to 84% KO efficiency in hPSCs.

## RESULTS

### modRNA-based delivery of CRISPR components can successfully knock out genes in hPSCs

To determine whether we could efficiently deliver Cas9 modRNA to hPSCs using lipofection, we synthesized Cas9-2A-GFP modRNA containing N1-methyl-pseudo-UTP (Hadas et al., 2019). 2A is a self-cleaving peptide that triggers ribosomal skipping along a single transcript during translation. Incorporation of the 2A linker within our Cas9-2A-GFP modRNA enables the protein synthesis of both Cas9 and GFP from a single modRNA. We transfected Cas9-2A-GFP into H1 and H9 cells. One day later, we quantified GFP expression using flow cytometry. We used a side scatter height (SSC-H) versus side scatter area (SSC-A) plot to exclude doublets for our flow cytometry data analyses (Figure S1A). We were able to achieve up to 90% transfection efficiency for Cas9-2A-GFP modRNA based on $GFP^+$ cells (Figure S1B). Next, to probe for the optimal amount of Cas9 modRNA and target-specific sgRNA, we made Cas9 modRNA without co-expression of GFP to knock out GFP from a human embryonic stem cell (hESC) OCT4-GFP reporter line (H1 OCT4-GFP) (Zwaka and Thomson, 2003). For designing our sgRNA targeting GFP, we chose to use the GFP sgRNA sequence reported by Sanjana et al. (2014). H1 OCT4-GFP cells were seeded in a 24-well plate and then transfected with different amounts of Cas9 modRNA and GFP sgRNA using Lipofectamine Stem

CellPress
OPEN ACCESS

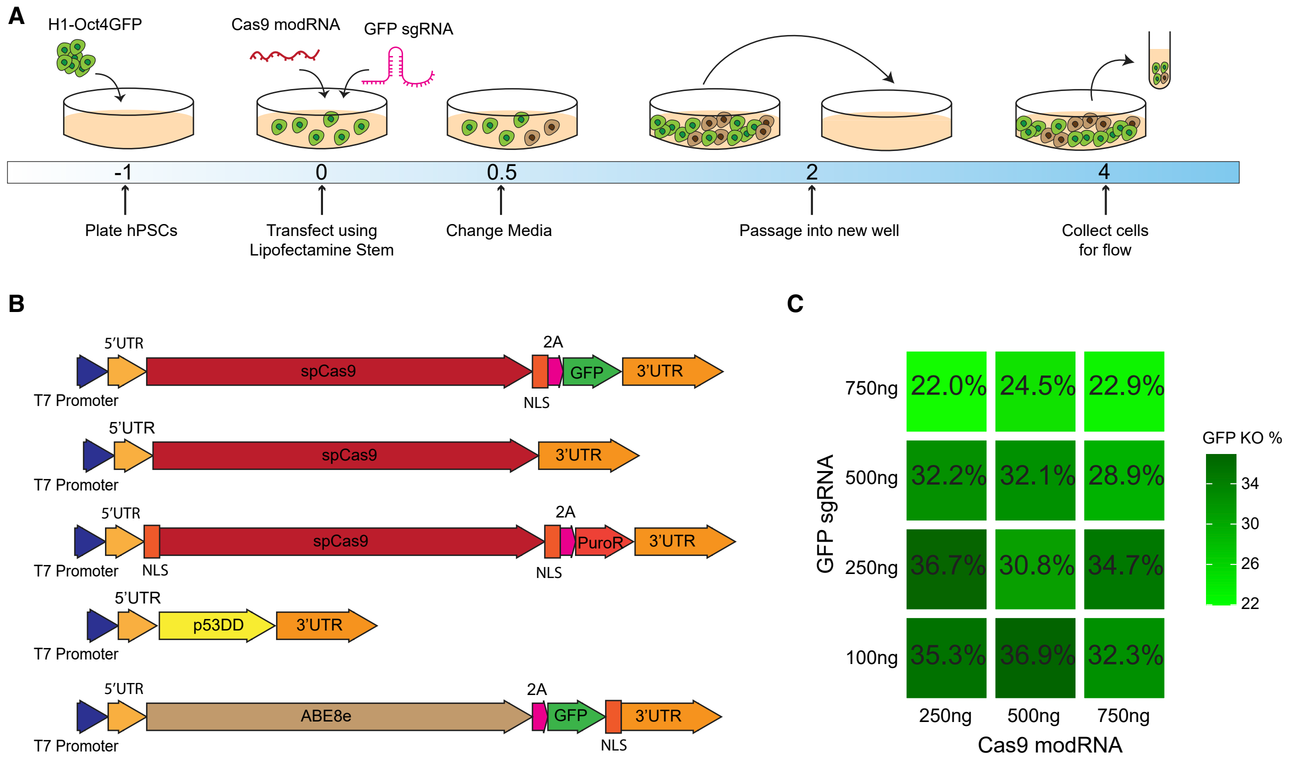


**Figure 1. Cas9 modRNA and sgRNA efficiently knock out an integrated GFP in hPSCs**

(A) Schematic diagram for knocking out GFP in H1 OCT4-GFP cells using Cas9 modRNA and *in vitro*-synthesized sgRNA.

(B) DNA templates used to synthesize modRNA for Cas9-2A-GFP, Cas9, Cas9-2A-Puro, p53DD, and ABE8e, including a summary of all the constructs used in this work.

(C) H1 OCT4-GFP cells were cultured on iMatrix-511 in mTeSR1 and transfected with different combinations of Cas9 modRNA and GFP sgRNA. On day 4, cells were collected, and GFP expression was analyzed via flow cytometry. The percentage of $GFP^{-}$ cells for each combination is shown in the form of a tiled heatmap. Experiments were repeated three times, and representative data are shown.

See also Figure S1.

transfection reagent (Figures 1A and 1B). Four days after transfection, cells were collected to quantify the percentage of $GFP^{-}$ cells using flow cytometry.

We tested various amounts of Cas9 modRNA (250, 500, or 750 ng) along with different doses of GFP sgRNA (100, 250, 500, and 750 ng) in H1 OCT4-GFP cells. We found that three Cas9 + sgRNA combinations (250 + 100, 250 + 250, and 500 + 100) achieved the highest KO efficiency (~36% $GFP^{-}$ cells on day 4) (Figures 1C and S1C). We also tested fewer amounts of Cas9 modRNA (125 or 250 ng) along with fewer doses of GFP sgRNA (10, 50, or 100 ng) but found that these conditions performed poorly when compared with our achieved three optimal combinations (Figure S1D). To minimize the total modRNA required for transfection, we decided to use the 250 ng Cas9 modRNA + 100 ng sgRNA combination for subsequent experiments.

## modRNA-based CRISPR system efficiently generates gene KOs in hPSCs

To investigate whether our modRNA-based CRISPR system was able to efficiently knock out genes in hPSCs, we decided to target *THY1* gene that encodes CD90 protein, a heavily glycosylated membrane protein that is expressed in undifferentiated hPSCs (Tang et al., 2011). We selected two potential sgRNA target sites for CD90 using ChopChop (Labun et al., 2019) (Figure 2A). We noticed that seeding the cells too sparsely for endogenous gene KO led to cell detachment and death. To tackle this, we decided to double our initial seeding density and include a Rho-associated kinase (ROCK) inhibitor (Vernardis et al., 2017) in our culture media, which led to better cell survival but reduced our transfection efficiency (Figure 2A). We found that CD90 sgRNA_1 was able to achieve higher KO efficiency than sgRNA_2 and therefore was used for all subsequent experiments (Figures 2B and S2A). Next, we wanted to see if we could improve CD90 KO efficiency via drug selection. We synthesized Cas9-2A-Puro (Cas9Puro) modRNA, which has a puromycin resistance gene linked to the Cas9 via a 2A linker (Figure 1B). Due to the larger size of the Cas9Puro construct, we also tested delivery of 300 ng Cas9Puro modRNA in addition to the previously determined 250 ng H9 cells that were seeded onto iMatrix-511-coated wells and transfected with either 250 ng Cas9, 250 ng Cas9Puro, or 300 ng Cas9-Puro modRNA. After 12 h, cells were treated with 1 μg/mL puromycin. After 24 h of drug selection, cells were stained with a TO-PRO 3 cell viability

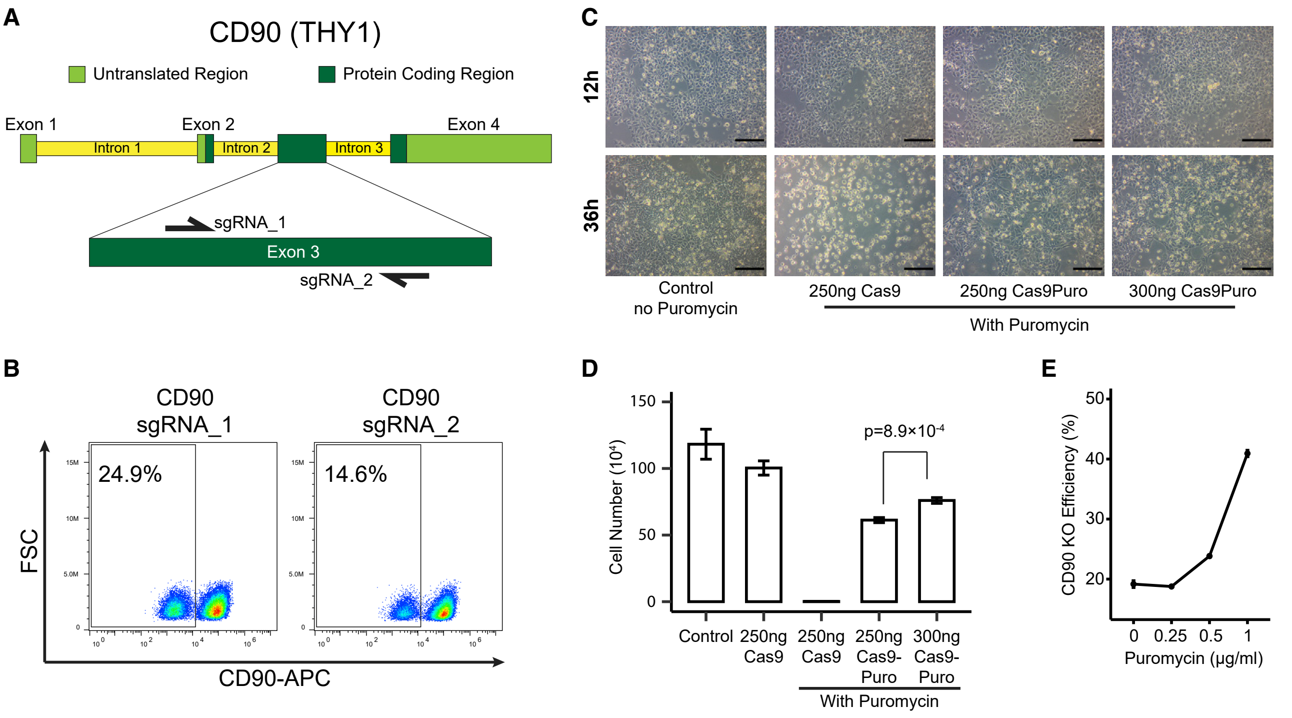


**Figure 2. Drug selection improved KO efficiency via Cas9Puro modRNA**

(A) Schematic of sgRNA design targeting *THY1* gene, encoding CD90 protein.

(B) H9 cells were cultured on iMatrix-511 in mTeSR1 and transfected with Cas9Puro modRNA and either the CD90_1 or CD90_2 sgRNA. On day 4, cells were collected, and CD90 expression was analyzed via flow cytometry. Representative flow cytometry results are shown for each target design.

(C) H9 cells were cultured on iMatrix-511 in mTeSR1 and transfected with either 300 or 250 ng Cas9Puro modRNA or 250 ng Cas9 modRNA. Transfected cells underwent drug selection for 24 h using puromycin beginning 12 h after transfection.

(D) Following drug selection, cells were imaged (scale bar, 200 µm) and stained using TO-PRO 3 cell viability reagent before being counted using a flow cytometer (n = 3; unpaired Student's t test).

(E) H9 cells were transfected with 300 ng Cas9Puro modRNA and 100 ng CD90_1 sgRNA and underwent 24 h of drug selection beginning 12 h after transfection. On day 5, cells were collected, and CD90 expression was analyzed by flow cytometry (n = 3).

See also Figure S2.

dye (excitation/emission 642/661 nm) and counted using a flow cytometer. As expected, treatment with puromycin effectively killed all cells in wells transfected with the Cas9 modRNA. However, in wells that were transfected with our Cas9Puro modRNA, we observed cell survival similar to our untreated control cells, indicating that our Cas9Puro modRNA could protect transfected cells from puromycin-mediated cell toxicity (Figures 2C, 2D, and S2B–S2D). Additionally, we observed consistently higher cell numbers in wells that were transfected with 300 ng Cas9Puro compared with 250 ng Cas9Puro, a difference that was statistically significant ($p = 8.9 \times 10^{-4}$, Student's t test) **(**Figure 2D). Due to the higher transfection efficiency using 300 ng Cas9Puro, as indicated by higher cell survival, we used 300 ng Cas9Puro modRNA for subsequent experiments. To evaluate whether puromycin treatment increases KO efficiency, we used our Cas9Puro modRNA to knock out CD90 in H9 cells accompanied by puromycin treatment at a concentration ranging from 0 to 1 µg/mL. We observed a greater than 2-fold increase in CD90 KO efficiency measured by the percentage of $CD90^-$ cells on day 5 post-transfection (Figure 2E).

## P53DD greatly increases modRNA-based genome-editing efficiency in hPSCs

While CRISPR-Cas9 systems have been used to engineer genomes of a wide variety of cell types, hPSCs have proven to be exceptionally difficult to engineer due to the toxicity of DSBs in these cells. Recently, Ihry et al. reported that the hPSC response to Cas9 induced DSBs is mediated by p53 (Ihry et al., 2018). Additionally, they showed that p53DD, a dominant negative mutant of p53, can transiently block p53 function and therefore reduce Cas9-induced toxicity in hPSCs. Therefore, we decided to synthesize p53DD modRNA to use with our modRNA-based Cas9 system. To compare modRNA and plasmid-mediated GFP KO in the presence or absence of p53DD, we transfected H1 OCT4-GFP cells with different combinations of plasmids or modRNAs (Figure 3A). For the plasmid-based method, hPSCs were transfected with a CRISPR plasmid (Jiang et al., 2022) expressing both Cas9 and sgRNA with or without a p53DD plasmid. For the modRNA-based method, hPSCs were transfected with Cas9Puro modRNA and sgRNA with or without p53DD modRNA. For the RNP method, hPSCs were transfected with Cas9 protein coupled with a sgRNA. For

CellPress
OPEN ACCESS

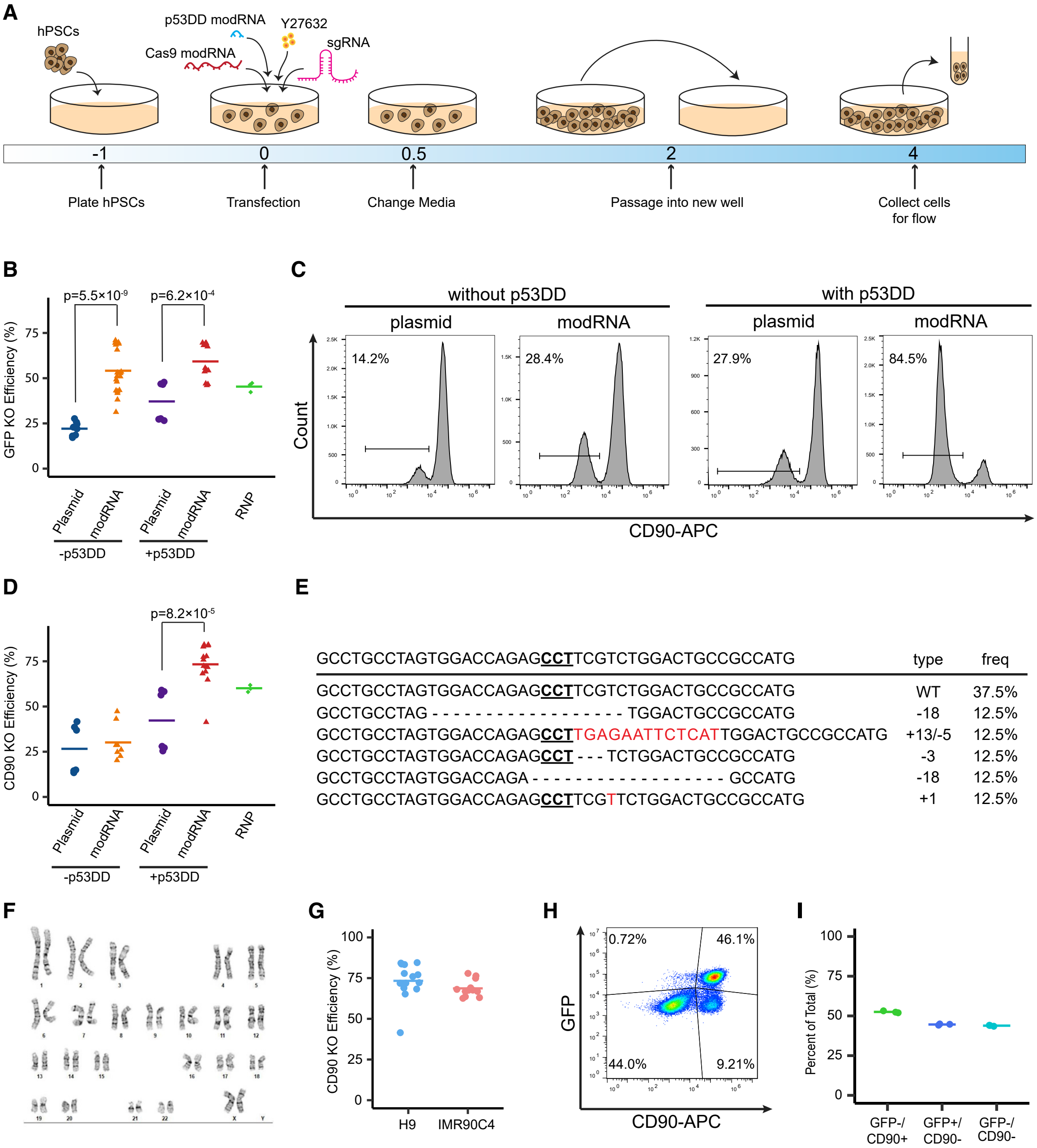


**Figure 3. P53DD modRNA increased Cas9 modRNA-mediated gene KO in hPSCs**

(A) Schematic of optimal transfection protocol with the addition of p53DD modRNA.

(B) Aggregated gene KO efficiencies across multiple replicates and batches in H1 OCT4-GFP cells, comparing results between transient plasmid DNA transfection and modRNA-based delivery with or without p53DD as well as RNP lipofection (plasmid: n = 9; modRNA: n = 20; plasmid + p53DD: n = 6; modRNA + p53DD: n = 13; RNP: n = 3).

(C) H9 cells cultured on iMatrix-511 in mTeSR1 were transiently transfected with either the plasmid DNA with or without p53DD plasmid, modRNA cocktail with or without p53DD modRNA, or Cas9 RNP. On day 5, cells were collected, and CD90 expression was analyzed via flow cytometry.

(D) Aggregated CD90 KO efficiencies across multiple replicates and batches in H9 cells, comparing results between transient plasmid DNA transfection and modRNA-based delivery without or with p53DD as well as RNP lipofection (plasmid: n = 6; modRNA: n = 8; plasmid + p53DD: n = 6; modRNA + p53DD: n = 14; RNP: n = 3; one-way ANOVA with post-hoc Tukey's test).

(E) Genotype of CD90KO H9 cells generated using CRISPR modRNA cocktail with p53DD modRNA (n = 8).

(F) G-banded karyotype analysis of CD90 KO H9 cells generated using modRNA cocktail with p53DD.

(G) IMR90C4 cells cultured on iMatrix-511 in mTeSR1 were transfected with Cas9Puro modRNA, CD90 sgRNA, and p53DD modRNA. On day 5, cells were collected, and CD90 expression was analyzed via flow cytometry (H9: n = 14; IMR90C4: n = 12).

*(legend continued on next page)*

GFP KO, we found that the modRNA method was superior to the plasmid method regardless of p53DD; modRNA with p53DD yielded the highest KO efficiency among these four conditions, and it was also better than the RNP method (Figures 3B and S3A). Next, we tested CD90 KO in H9 cells with these 5 conditions and found that the modRNA with p53DD method achieved the highest CD90 KO efficiency, yielding 73.3% ± 11.2% KO efficiency (Figures 3C, 3D, and S3B). Moreover, we tested our modRNA method by knocking out the Wnt signaling effector protein β-catenin. For synthesizing β-catenin sgRNA, we used the target sequence reported before (Jiang et al., 2022). The modRNA with p53DD method achieved the highest β-catenin KO efficiency among five conditions (Figures S3C–S3E). The RNP method achieved minimal β-catenin KO efficiency using this sgRNA (Figures S3C–S3E), indicating that the RNP method may exhibit greater variations in knocking out different genes. Furthermore, the RNP method yielded fewer cells than the modRNA method (Figure S3F).

Next, we characterized the Cas9 cleavage sites using the TOPO-TA cloning method with CD90 KO cells. We observed a diverse variety of genome-editing types in our CD90 KO cells with both insertion and deletion mutations (Figure 3E). To determine whether monoallelic targeting is likely to occur, we compared mean fluorescent intensity (MFI) of CD90 in the CD90$^+$ population between un-transfected cells (control) and cells transfected with Cas9 modRNA and CD90 sgRNA (CD90 KO). The MFI in the control sample is higher than the CD90 KO sample, indicating that monoallelic targeting may occur in the CD90 KO samples (Figure S3G). We also analyzed three potential off-target locations and did not observe any off-target mutations (Figure S3H). Furthermore, hPSCs edited with our modRNA with p53DD method maintained normal karyotype (Figure 3F).

For modRNA-based gene editing in induced pluripotent stem cells (iPSCs), we compared CD90 KO in H9 cells and IMR90C4 iPSCs and found that our modRNA with p53DD method was equally effective in editing iPSCs, with a KO efficiency of 68.7% ± 5.1% (Figures 3G and S3I). Similarly, we demonstrated that our modRNA with p53DD method generated β-catenin KO at a similar efficiency in iPSCs as in H9 cells (Figure S3J).

Next, we decided to examine whether our modRNA-based method could simultaneously target multiple genomic sites and thus knock out multiple genes. We seeded our H1 OCT4-GFP cells and transfected them with Cas9Puro modRNA, GFP sgRNA, CD90 sgRNA, and p53DD modRNA. We collected cells on day 5 post-transfection to quantify GFP and CD90 expression using flow cytometry. We observed 43.9% ± 0.3% of cells that were deficient in both GFP and CD90 expression after one single transfection (Figures 3H and 3I).

Eukaryotic RNA is normally capped at the 5′ end with 7-methylguanosine (m7G), commonly referred to as cap 0 structure, and is important for translational initiation and prevents degradation of the mRNA transcript. When synthesizing modRNA, the cap 0 structure is introduced by the addition of the anti-reverse cap analog (ARCA) to the *in vitro* transcription reaction mix. Higher-order eukaryotes will instead have a cap 1 structure, in which the first nucleotide proximal to the cap structure is methylated. Using modRNA with the cap 1 modification can potentially further abrogate the innate immune response compared with cap 0 due to its reduced affinity for binding RIG-I, MDA5, and IFIT-1 (Abbas et al., 2017; Devarkar et al., 2016; Rehwinkel and Gack, 2020; Vaidyanathan et al., 2018; Züst et al., 2011). To synthesize modRNA with the cap 1 modification, we used site-directed mutagenesis to convert the G to an A proximal to the T7 promotor sequence in modRNA cap 0 (modRNAc0) plasmid, yielding a modRNAc1 plasmid. Then, we cloned our Cas9Puro insert into modRNAc1 plasmid. In addition, we replaced the ARCA reagent with the CleanCap AG reagent. Our data showed that both cap 0 and cap 1 modRNA could efficiently knock out CD90 in hPSCs (Figure S4), indicating that the reduced immunogenicity of cap 1 modRNA did not further improve gene KO efficiency in hPSCs.

## ABE8e modRNA outperforms its plasmid counterpart for genome editing in hPSCs

Besides Cas9, base editing can introduce single-nucleotide variants into the genome and represents another important technique for genome editing. The adenosine base editor ABE8e was our base editor of choice (Richter et al., 2020). To determine if base-editing efficiencies using modRNA could outperform plasmid-based delivery, we decided to knock out the *B2M* gene, a protein subunit required for surface expression of all class I major histocompatibility complex molecules. Our B2M KO strategy employed base editing of the splice donor site, thus rendering the spliceosome incapable of splicing the transcript correctly and deactivating it (Figure 4A). Using the SpliceR program (Kluesner et al., 2021), we chose the most efficient sgRNA for B2M KO using the ABE8e system. Next, hPSCs were transfected with ABE8e, which was either encoded by a plasmid or by modRNA, and a sgRNA targeting the splice donor site of intron 1. The plasmid delivery was conducted in two different mass ratios of the ABE8e (Data S1) to sgRNA plasmid (1:1 and 3:1). ABE8e-mediated B2M KO efficiencies were then measured using flow cytometry for B2M expression 5 days post-transfection. Whereas the plasmid-based method achieved 16.1% ± 0.8% and 12.3% ± 2.2% KO efficiencies (1:1 and 3:1 mass ratio, respectively), our modRNA-based method generated a much higher KO efficiency (69.6% ± 3.8%) (Figures 4B and 4C). To ensure that the lack of B2M expression was the result of edited splice donor, we opted to characterize intron 1 splice donor site in a B2M KO clone via the TOPO-TA cloning method. We found that both alleles

(H and I) H1 OCT4-GFP cells were cultured on iMatrix-511 in mTeSR1 using a 12-well plate and transfected with 1,200 ng Cas9Puro modRNA, 200 ng CD90_1 sgRNA, 200 ng GFP sgRNA, and 200 ng p53DD modRNA. On day 5, cells were collected, and GFP/CD90 expression was analyzed via flow cytometry (n = 3).
(H) Representative flow cytometry plot from day 5.
(I) Quantification of flow cytometry results from day 5 cells.
See also Figures S3 and S4.

CellPress
OPEN ACCESS

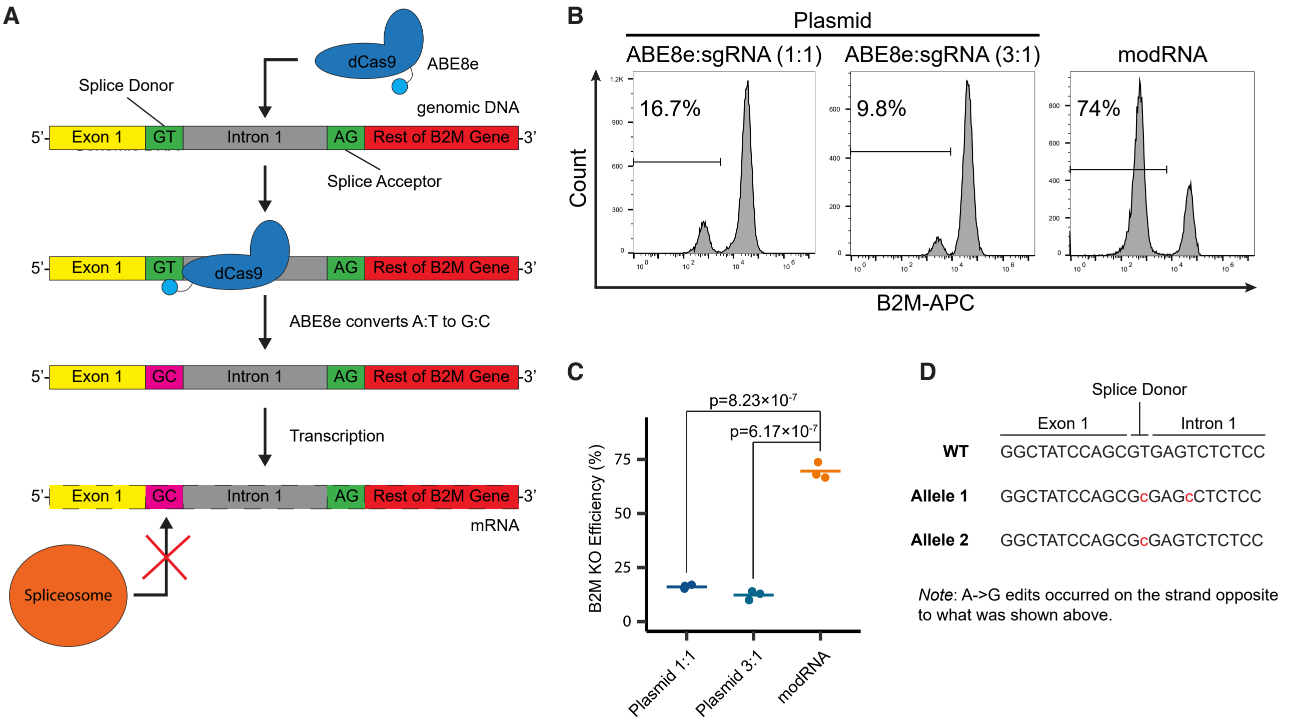


**Figure 4. modRNA ABE8e is more efficient over plasmid-based method**

(A) Schematic of mechanism for gene KO via base editing. The dCas9 guides the fused ABE8e to the specific genomic region to perform the desired base edit. This desired base edit mutates the splice acceptor or donor region so that after transcription, the spliceosome fails to splice out the intron or splices an exon, respectively.

(B) Representative flow cytometry plots of cell population that were transfected with ABE8e + sgRNA, which were delivered in plasmid DNA or modRNA form. Cell populations were stained with a conjugated anti-B2M-APC antibody.

(C) Quantification of $B2M^{-}$ cells following either plasmid DNA or modRNA ABE8e transfection (n = 3; one-way ANOVA with post-hoc Tukey's test).

(D) Sequencing result of the B2M intron 1 splice donor site within a single cell clonal line. This indicates that one allele had two A:T to G:C base edits (both within the ABE8e editing window) and the other allele received only the desired splice donor base edit (base edits shown in red font).

possessed the desired A:T to G:C editing at the splice donor site (Figure 4D). One allele also had a second base edit, 4 base pairs away within the intron, because this site is still within the ABE8e's base-editing window. Overall, our experiments demonstrated that our modRNA-based ABE8e system is about four times more efficient than its plasmid counterpart at generating base edits and enabling gene KO in hPSCs.

## DISCUSSION

Our research outlines methods for efficient CRISPR-mediated gene KOs in hPSCs using a modRNA-based Cas9 or ABE8e system, which can be widely adopted for most labs without requiring electroporation or nucleofection devices. We tested the efficacy of our modRNA Cas9 system using multiple hPSC lines, including two hESC lines as well as a human iPSC line, demonstrating the general applicability. Our approach is highly flexible to a variety of experimental conditions owing to the Cas9Puro modRNA, which can be used in conjunction with puromycin treatment to increase KO efficiency when high transfection efficiency is not possible for certain cell types. Integration of the p53DD modRNA into our system significantly increases gene KO efficiency by reducing Cas9 induced DSB toxicity in hPSCs.

We also studied B2M KO in hPSCs via inactivation of the splice donor using the ABE8e base editor. We found that the modRNA ABE8e method is more efficient compared with the plasmid format. The main advantage of using base editors for generating gene KO in hPSCs is the elimination of DSBs generated by Cas9. This abolishes the undesired chromosomal rearrangements that result from DSBs and lowers the chances of detrimental off-target indels, thus providing a more clinically relevant genome engineering tool for hPSCs. Our modRNA ABE8e method had a KO efficiency similar to that of our Cas9 with p53DD modRNA method (69.6% ± 3.8% versus 73.3% ± 11.2%), highlighting its potential as an alternative to CRISPR-Cas9-based strategies.

In summary, we demonstrated that when CRISPR-Cas9 with p53DD or ABE8e modRNA is transfected into hPSCs, it outperforms the plasmid-based method. The increased efficiency of modRNA methods is likely due to higher transfection efficiencies and higher Cas9 or ABE8e protein expression levels in the hPSCs. Since it is not 100% efficient, as is the case with other delivery methods, clonal isolation is still required for some downstream gene KO studies. Despite this, our Cas9 with p53DD or ABE8e modRNA method results in extremely high transfection efficiency and very high Cas9 or

ABE8e expression levels, ultimately generating higher KO efficiencies in hPSCs.

### Limitations of the study

There are limitations inherent to our modRNA-based CRISPR method. First, we used wild-type SpCas9 for genome editing which has a protospacer adjacent motif (PAM) NGG. Thus, due to PAM NGG restriction, our method limits target site recognition to a subset of sequences in the genome. To remove this constraint, we may use modRNA expression of engineered near-PAMless Cas9 (Walton et al., 2020). Second, gene KO performance with CRISPR systems is inherently tied to the sgRNA used. When applying our methods for gene KOs, multiple sgRNAs are needed for testing their on- and off-target editing efficiencies. In addition, in this study, we used the TOPO-TA cloning method to identify on- and off-target editing. However, TOPO-TA cloning may not be sensitive enough. Thus, in the future, using next-generation sequencing (NGS) and GUIDE-seq or Digenome-seq may be needed to quantify editing before any edited stem cells are used for therapies. Furthermore, our modRNA-based CRISPR system transfection and editing efficiency has not reached 100%, and single-cell clone isolation is still needed for isolating KO clones. Development of new transfection reagents and/or inclusion of small molecules targeting DNA repair pathways may further improve modRNA-based gene-editing performance (Riesenberg and Maricic, 2018).

## STAR★METHODS

Detailed methods are provided in the online version of this paper and include the following:

- KEY RESOURCES TABLE
- RESOURCE AVAILABILITY
  - Lead contact
  - Materials availability
  - Data and code availability
- EXPERIMENTAL MODEL AND SUBJECT DETAILS
  - Cell lines
- METHOD DETAILS
  - Maintenance of hPSCs
  - Modified mRNA (modRNA) synthesis
  - sgRNA synthesis
  - Transfection of Cas9 modRNA or plasmid into hPSCs
  - Transfection of ABE8e modRNA or plasmid into hPSCs
  - Flow cytometry
  - TOPO TA cloning for sequencing
- QUANTIFICATION AND STATISTICAL ANALYSIS

## SUPPLEMENTAL INFORMATION

Supplemental information can be found online at https://doi.org/10.1016/j.crmeth.2022.100290.

## ACKNOWLEDGMENTS

This work was supported by NIH NIBIB R21EB026035 (to X.L.L.), NIH NIAMS R01 AR072731 (to J.Y.), NSF CBET-1943696 (to X.L.L.), NSF CBET-2143064 (to X.B.), and Penn State startup funding (to X.L.L.).

## AUTHOR CONTRIBUTIONS

T.H., A.H., and X.L.L. designed the experiments and analyzed the results. T.H., A.H., and Y.J. performed the experiments and analyzed data. T.H., A.H., and X.L.L. wrote the manuscript. J.Y., X.B., and X.L.L. contributed to the revision of the manuscript. X.L.L. provided funding and supervised the experiments.

## DECLARATION OF INTERESTS

X.L.L., T.H., A.H., and Y.J. are listed as inventors on a provisional patent application (no. 63/335,568), which was filed on April 27, 2022. The other authors declare no competing interests.

Received: March 3, 2022
Revised: July 9, 2022
Accepted: August 16, 2022
Published: September 7, 2022

## REFERENCES

Abbas, Y.M., Laudenbach, B.T., Martínez-Montero, S., Cencic, R., Habjan, M., Pichlmair, A., Damha, M.J., Pelletier, J., and Nagar, B. (2017). Structure of human IFIT1 with capped RNA reveals adaptable mRNA binding and mechanisms for sensing N1 and N2 ribose 2?-O methylations. Proc. Natl. Acad. Sci. USA *114*, E2106–E2115. https://doi.org/10.1073/pnas.1612444114.

Antoniou, P., Miccio, A., and Brusson, M. (2021). Base and prime editing technologies for blood disorders. Front. Genome Ed. *3*, 618406. https://doi.org/10.3389/fgeed.2021.618406.

Cong, L., Ran, F.A., Cox, D., Lin, S., Barretto, R., Habib, N., Hsu, P.D., Wu, X., Jiang, W., Marraffini, L.A., and Zhang, F. (2013). Multiplex genome engineering using CRISPR/Cas systems. Science *339*, 819–823. https://doi.org/10.1126/science.1231143.

Devarkar, S.C., Wang, C., Miller, M.T., Ramanathan, A., Jiang, F., Khan, A.G., Patel, S.S., and Marcotrigiano, J. (2016). Structural basis for m7G recognition and 2′-O-methyl discrimination in capped RNAs by the innate immune receptor RIG-I. Proc. Natl. Acad. Sci. USA *113*, 596–601. https://doi.org/10.1073/pnas.1515152113.

Gaudelli, N.M., Komor, A.C., Rees, H.A., Packer, M.S., Badran, A.H., Bryson, D.I., and Liu, D.R. (2017). Programmable base editing of AT to GC in genomic DNA without DNA cleavage. Nature *551*, 464–471. https://doi.org/10.1038/nature24644.

Gaudelli, N.M., Lam, D.K., Rees, H.A., Solá-Esteves, N.M., Barrera, L.A., Born, D.A., Edwards, A., Gehrke, J.M., Lee, S.J., Liquori, A.J., et al. (2020). Directed evolution of adenine base editors with increased activity and therapeutic application. Nat. Biotechnol. *38*, 892–900. https://doi.org/10.1038/S41587-020-0491-6.

Hadas, Y., Sultana, N., Youssef, E., Sharkar, M.T.K., Kaur, K., Chepurko, E., and Zangi, L. (2019). Optimizing modified mRNA in vitro synthesis protocol for heart gene therapy. Mol. Ther. Methods Clin. Dev. *14*, 300–305. https://doi.org/10.1016/J.OMTM.2019.07.006.

Hsu, M.N., Chang, Y.H., Truong, V.A., Lai, P.L., Nguyen, T.K.N., and Hu, Y.C. (2019). CRISPR technologies for stem cell engineering and regenerative medicine. Biotechnol. Adv. *37*, 107447. https://doi.org/10.1016/j.biotechadv.2019.107447.

Ihry, R.J., Worringer, K.A., Salick, M.R., Frias, E., Ho, D., Theriault, K., Kommineni, S., Chen, J., Sondey, M., Ye, C., et al. (2018). p53 inhibits CRISPR-Cas9 engineering in human pluripotent stem cells. Nat. Med. *24*, 939–946. https://doi.org/10.1038/s41591-018-0050-6.

Jiang, T., Henderson, J.M., Coote, K., Cheng, Y., Valley, H.C., Zhang, X.O., Wang, Q., Rhym, L.H., Cao, Y., Newby, G.A., et al. (2020). Chemical modifications of adenine base editor mRNA and guide RNA expand its application scope. Nat. Commun. *11*, 1979. https://doi.org/10.1038/S41467-020-15892-8.

Jiang, Y., Chen, C., Randolph, L.N., Ye, S., Zhang, X., Bao, X., and Lian, X.L. (2021). Generation of pancreatic progenitors from human pluripotent stem

CellPress
OPEN ACCESS

cells by small molecules. Stem Cell Rep. *16*, 2395–2409. https://doi.org/10.1016/j.stemcr.2021.07.021.

Jiang, Y., Hoenisch, R.C., Chang, Y., Bao, X., Cameron, C.E., and Lian, X.L. (2022). Robust genome and RNA editing via CRISPR nucleases in PiggyBac systems. Bioact. Mater. *14*, 313–320. https://doi.org/10.1016/J.BIOACTMAT.2022.01.046.

Jinek, M., Chylinski, K., Fonfara, I., Hauer, M., Doudna, J.A., and Charpentier, E. (2012). A programmable dual-RNA – guided DNA endonuclease in adaptive bacterial immunity. Science *337*, 816–821. https://doi.org/10.1126/science.1225829.

Karikó, K., Buckstein, M., Ni, H., and Weissman, D. (2005). Suppression of RNA recognition by Toll-like receptors: the impact of nucleoside modification and the evolutionary origin of RNA. Immunity *23*, 165–175. https://doi.org/10.1016/J.IMMUNI.2005.06.008.

Karikó, K., Muramatsu, H., Welsh, F.A., Ludwig, J., Kato, H., Akira, S., and Weissman, D. (2008). Incorporation of pseudouridine into mRNA yields superior nonimmunogenic vector with increased translational capacity and biological stability. Mol. Ther. *16*, 1833–1840. https://doi.org/10.1038/mt.2008.200.

Kluesner, M.G., Lahr, W.S., Lonetree, C.L., Smeester, B.A., Qiu, X., Slipek, N.J., Claudio Vázquez, P.N., Pitzen, S.P., Pomeroy, E.J., Vignes, M.J., et al. (2021). CRISPR-Cas9 cytidine and adenosine base editing of splice-sites mediates highly-efficient disruption of proteins in primary and immortalized cells. Nat. Commun. *12*, 2437–2512. https://doi.org/10.1038/s41467-021-22009-2.

Koblan, L.W., Doman, J.L., Wilson, C., Levy, J.M., Tay, T., Newby, G.A., Maianti, J.P., Raguram, A., and Liu, D.R. (2018). Improving cytidine and adenine base editors by expression optimization and ancestral reconstruction. Nat. Biotechnol. *36*, 843–846. https://doi.org/10.1038/NBT.4172.

Komor, A.C., Kim, Y.B., Packer, M.S., Zuris, J.A., and Liu, D.R. (2016). Programmable editing of a target base in genomic DNA without double-stranded DNA cleavage. Nature *533*, 420–424. https://doi.org/10.1038/NATURE17946.

Labun, K., Montague, T.G., Krause, M., Torres Cleuren, Y.N., Tjeldnes, H., and Valen, E. (2019). CHOPCHOP v3: expanding the CRISPR web toolbox beyond genome editing. Nucleic Acids Res. *47*, W171–W174. https://doi.org/10.1093/nar/gkz365.

Lian, X., Bao, X., Al-Ahmad, A., Liu, J., Wu, Y., Dong, W., Dunn, K.K., Shusta, E.V., and Palecek, S.P. (2014). Efficient differentiation of human pluripotent stem cells to endothelial progenitors via small-molecule activation of WNT signaling. Stem Cell Rep. *3*, 804–816. https://doi.org/10.1016/J.STEMCR.2014.09.005.

Lian, X., Bao, X., Zilberter, M., Westman, M., Fisahn, A., Hsiao, C., Hazeltine, L.B., Dunn, K.K., Kamp, T.J., and Palecek, S.P. (2015). Chemically defined, albumin-free human cardiomyocyte generation. Nat. Methods *12*, 595–596. https://doi.org/10.1038/nmeth.3448.

Lian, X., Hsiao, C., Wilson, G., Zhu, K., Hazeltine, L.B., Azarin, S.M., Raval, K.K., Zhang, J., Kamp, T.J., and Palecek, S.P. (2012). Robust cardiomyocyte differentiation from human pluripotent stem cells via temporal modulation of canonical Wnt signaling. Proc. Natl. Acad. Sci. USA *109*, E1848–E1857. https://doi.org/10.1073/PNAS.1200250109/-/DCSUPPLEMENTAL.

Lian, X., Zhang, J., Azarin, S.M., Zhu, K., Hazeltine, L.B., Bao, X., Hsiao, C., Kamp, T.J., and Palecek, S.P. (2013). Directed cardiomyocyte differentiation from human pluripotent stem cells by modulating Wnt/β-catenin signaling under fully defined conditions. Nat. Protoc. *8*, 162–175. https://doi.org/10.1038/nprot.2012.150.

Liu, Z., Hui, Y., Shi, L., Chen, Z., Xu, X., Chi, L., Fan, B., Fang, Y., Liu, Y., Ma, L., et al. (2016). Efficient CRISPR/Cas9-Mediated versatile, predictable, and donor-free gene knockout in human pluripotent stem cells. Stem Cell Rep. *7*, 496–507. https://doi.org/10.1016/j.stemcr.2016.07.021.

Mali, P., Yang, L., Esvelt, K.M., Aach, J., Guell, M., DiCarlo, J.E., Norville, J.E., and Church, G.M. (2013). RNA-guided human genome engineering via Cas9. Science *339*, 823–826. https://doi.org/10.1126/science.1232033.

Martin, R.M., Ikeda, K., Cromer, M.K., Uchida, N., Nishimura, T., Romano, R., Tong, A.J., Lemgart, V.T., Camarena, J., Pavel-Dinu, M., et al. (2019). Highly efficient and marker-free genome editing of human pluripotent stem cells by CRISPR-cas9 RNP and AAV6 donor-mediated homologous recombination. Cell Stem Cell *24*, 821–828.e5. https://doi.org/10.1016/j.stem.2019.04.001.

Rehwinkel, J., and Gack, M.U. (2020). RIG-I-like receptors: their regulation and roles in RNA sensing. Nat. Rev. Immunol. *20*, 537–551. https://doi.org/10.1038/s41577-020-0288-3.

Richter, M.F., Zhao, K.T., Eton, E., Lapinaite, A., Newby, G.A., Thuronyi, B.W., Wilson, C., Koblan, L.W., Zeng, J., Bauer, D.E., et al. (2020). Phage-assisted evolution of an adenine base editor with improved Cas domain compatibility and activity. Nat. Biotechnol. *38*, 883–891. https://doi.org/10.1038/s41587-020-0453-z.

Riesenberg, S., and Maricic, T. (2018). Targeting repair pathways with small molecules increases precise genome editing in pluripotent stem cells. Nat. Commun. *9*, 2164. https://doi.org/10.1038/S41467-018-04609-7.

Sanjana, N.E., Shalem, O., and Zhang, F. (2014). Improved vectors and genome-wide libraries for CRISPR screening. Nat. Methods *11*, 783–784. https://doi.org/10.1038/nmeth.3047.

Sternberg, S.H., Lafrance, B., Kaplan, M., and Doudna, J.A. (2015). Conformational control of DNA target cleavage by CRISPR-Cas9. Nature *527*, 110–113. https://doi.org/10.1038/NATURE15544.

Suknuntha, K., Tao, L., Brok-Volchanskaya, V., D'Souza, S.S., Kumar, A., and Slukvin, I. (2018). Optimization of synthetic mRNA for highly efficient translation and its application in the generation of endothelial and hematopoietic cells from human and primate pluripotent stem cells. Stem Cell Rev. Rep. *14*, 525–534. https://doi.org/10.1007/S12015-018-9805-1.

Sürün, D., Schneider, A., Mircetic, J., Neumann, K., Lansing, F., Paszkowskir-ogacz, M., Hänchen, V., Leekirsch, M.A., and Buchholz, F. (2020). Efficient generation and correction of mutations in human iPS cells utilizing mRNAs of CRISPR base editors and prime. Genes *11*, E511. https://doi.org/10.3390/genes11050511.

Svitkin, Y.V., Cheng, Y.M., Chakraborty, T., Presnyak, V., John, M., and Sonenberg, N. (2017). N1-methyl-pseudouridine in mRNA enhances translation through eIF2$\alpha$-dependent and independent mechanisms by increasing ribosome density. Nucleic Acids Res. *45*, 6023–6036. https://doi.org/10.1093/NAR/GKX135.

Tang, C., Lee, A.S., Volkmer, J.P., Sahoo, D., Nag, D., Mosley, A.R., Inlay, M.A., Ardehali, R., Chavez, S.L., Pera, R.R., et al. (2011). An antibody against SSEA-5 glycan on human pluripotent stem cells enables removal of teratoma-forming cells. Nat. Biotechnol. *29*, 829–834. https://doi.org/10.1038/nbt.1947.

Vaidyanathan, S., Azizian, K.T., Haque, A.K.M.A., Henderson, J.M., Hendel, A., Shore, S., Antony, J.S., Hogrefe, R.I., Kormann, M.S.D., Porteus, M.H., and McCaffrey, A.P. (2018). Uridine depletion and chemical modification increase Cas9 mRNA activity and reduce immunogenicity without HPLC purification. Mol. Ther. Nucleic Acids *12*, 530–542. https://doi.org/10.1016/j.omtn.2018.06.010.

Vernardis, S.I., Terzoudis, K., Panoskaltsis, N., and Mantalaris, A. (2017). Human embryonic and induced pluripotent stem cells maintain phenotype but alter their metabolism after exposure to ROCK inhibitor. Sci. Rep. *7*, 42138–42211. https://doi.org/10.1038/srep42138.

Walton, R.T., Christie, K.A., Whittaker, M.N., and Kleinstiver, B.P. (2020). Unconstrained genome targeting with near-PAMless engineered CRISPR-Cas9 variants. Science *368*, 290–296. https://doi.org/10.1126/SCIENCE.ABA8853.

Wu, X., Dao Thi, V.L., Huang, Y., Billerbeck, E., Saha, D., Hoffmann, H.H., Wang, Y., Silva, L.A.V., Sarbanes, S., Sun, T., et al. (2018). Intrinsic immunity shapes viral resistance of stem cells. Cell *172*, 423–438.e25. https://doi.org/10.1016/J.CELL.2017.11.018.

Xu, J., Zhou, C., Foo, K.S., Yang, R., Xiao, Y., Bylund, K., Sahara, M., and Chien, K.R. (2020). Genome-wide CRISPR screen identifies ZIC2 as an essential gene that controls the cell fate of early mesodermal precursors to human heart progenitors. Stem Cell. *38*, 741–755. https://doi.org/10.1002/STEM.3168.

Yilmaz, A., Peretz, M., Aharony, A., Sagi, I., and Benvenisty, N. (2018). Defining essential genes for human pluripotent stem cells by CRISPR–Cas9 screening

in haploid cells. Nat. Cell Biol. *20*, 610–619. https://doi.org/10.1038/s41556-018-0088-1.

Züst, R., Cervantes-Barragan, L., Habjan, M., Maier, R., Neuman, B.W., Ziebuhr, J., Szretter, K.J., Baker, S.C., Barchet, W., Diamond, M.S., et al. (2011). Ribose 2’-O-methylation provides a molecular signature for the distinction of self and non-self mRNA dependent on the RNA sensor Mda5. Nat. Immunol. *12*, 137–143. https://doi.org/10.1038/ni.1979.

Zwaka, T.P., and Thomson, J.A. (2003). Homologous recombination in human embryonic stem cells. Nat. Biotechnol. *21*, 319–321. https://doi.org/10.1038/nbt788.

CellPress
OPEN ACCESS

## STAR★METHODS

### KEY RESOURCES TABLE

| REAGENT or RESOURCE | SOURCE | IDENTIFIER |
|---|---|---|
| Antibodies | | |
| B2M-APC | Biolegend | 316312; RRID:AB_10641281 |
| CD90-APC | Biolegend | 328113; RRID:AB_893440 |
| β-Catenin | BD | 610153; RRID:AB_397554 |
| Goat anti-mouse IgG, Alexa Fluor 647 | Thermo Fisher Scientific | A-21235; RRID:AB_2535804 |
| Recombinant DNA | | |
| PB-CRISPR | Addgene | 160047 |
| pCE-mp53DD | Addgene | 41856 |
| pGuide (for DNA plasmid gRNA delivery) | Addgene | 64711 |
| XLoneV3-ABE8e | This paper | Pending submission |
| modRNAc0-Cas9 | This paper | Addgene 170180 |
| modRNAc0-Cas9-2A-GFP | This paper | Addgene 170181 |
| modRNAc0-Cas9-2A-Puro | This paper | Addgene 172855 |
| modRNAc0-p53DD | This paper | Addgene 176902 |
| modRNAc0-ABE8e | This paper | Addgene 178177 |
| Chemicals, peptides, and recombinant proteins | | |
| iMatrix-511 | Iwai North America Inc | N-892021 |
| mTeSR1 | StemCell Technologies | 85850 |
| Accutase | Innovative Cell Technologies | AT104500 |
| DMEM | Thermo Fisher Scientific | 11965118 |
| Y-27632 | Selleck Chemicals | 146986-50-7 |
| N1-methyl-pseudo-UTP | TriLink Biotechnologies | N-1081 |
| Anti-Reverse Cap Analog (ARCA) | TriLink Biotechnologies | N-7003 |
| CleanCap AG | TriLink Biotechnologies | N-7113 |
| Lipofectamine Stem Transfection Reagent | Thermo Fisher Scientific | STEM00015 |
| Opti-MEM Reduced Serum Medium | Thermo Fisher Scientific | 51985034 |
| Doxycycline | Sigma-Aldrich | D9891 |
| PBS | Thermo Fisher Scientific | 14190250 |
| Bovine Serum Albumin | VWR | 10842-692 |
| GoTaq G2 Hot Start Master Mix | Promega | M7422 |
| LB Broth medium | Thermo Fisher Scientific | 10855001 |
| Puromycin | Thermo Fisher Scientific | A1113803 |
| Critical commercial assays | | |
| ZymoClean Gel DNA Recovery kit | Zymo Research | D4001 |
| In-Fusion HD Cloning Plus CE kit | Takara Bio | 638916 |
| DNA Clean & Concentrator-5 | Zymo Research | D4004 |
| MEGAscript T7 Transcription kit | Thermo Fisher Scientific | AM1334 |
| MEGAclear transcription clean-up kit | Thermo Fisher Scientific | AM1908 |
| EnGen sgRNA Synthesis kit, *S. pyogenes* | NEB | E3322 |
| Zymo Quick DNA Miniprep Plus kit | Zymo Research | D4068 |
| TOPO TA Cloning Kit for Sequencing | Thermo Fisher Scientific | 45-003-0 |
| Zyppy Plasmid Miniprep Kit | Zymo Research | D4020 |
| Experimental models: Cell lines | | |
| Human: H9 hESCs | WiCell | WB0299 |
| Human: H1 OCT4-GFP hESCs | WiCell | MCB-01 |
| Human: IMR90C4 iPSCs | WiCell | WB65317 |

(*Continued on next page*)

| *Continued* | | |
|---|---|---|
| REAGENT or RESOURCE | SOURCE | IDENTIFIER |
| Software and algorithms | | |
| FlowJo | http://www.flowjo.com/ | N/A |
| Oligonucleotides | | |
| For gene cloning, sequencing editing sites, and gRNA sequences. | This paper | See Table S1 |

## RESOURCE AVAILABILITY

### Lead contact

Further information and requests for resources and reagents should be directed to and will be fulfilled by the lead contact Dr. Xiaojun Lance Lian (Lian@psu.edu).

### Materials availability

All plasmids generated from this paper will be available at addgene.

### Data and code availability

- The published article includes all the dataset generated during this study.
- This paper does not report original code.
- Any additional information required to re-analyze the data reported in this paper is available from the lead contact upon request.

## EXPERIMENTAL MODEL AND SUBJECT DETAILS

### Cell lines

Three pluripotent cell lines, H9, H1 OCT4-GFP, and IMR90C4, were used for this study. These lines were obtained from WiCell Research Institute. All cell culture experiments involving human pluripotent stem cell lines were approved by the Embryonic Stem Cell Oversight Committee at the Pennsylvania State University and carried out in accordance with the approved guidelines.

## METHOD DETAILS

### Maintenance of hPSCs

hPSCs were maintained on iMatrix-511 (Iwai North America) coated plates in mTeSR1 medium (STEMCELL Technologies). Cells were regularly passaged when they reached 80–90% confluency, usually 3–4 days after the previous passage. For passaging, cell medium was aspirated and 1mL of Accutase (Innovative Cell Technologies) was added to each well. Cells were incubated at 37°C, 5% $CO_2$ for 5 to 10 min. Dissociated cells were transferred to excess DMEM at a 1:2 (vol/vol) ratio and centrifuged at 1000 rpm for 4 min. New wells were precoated with 0.75 μg/mL iMatrix-511 and incubated at 37°C, 5% $CO_2$ for 10 min. After centrifugation, cell pellet was resuspended in mTeSR1 with 5 μM Y-27632 (Selleck Chemicals). 10,000–20,000 cells/$cm^2$ were seeded onto iMatrix-511 coated wells. For regular maintenance cells were cultured in six-well plates.

### Modified mRNA (modRNA) synthesis

Cas9-2A-GFP, Cas9, Cas9Puro, p53DD, and ABE8e template DNA was PCR amplified from the donor plasmid using appropriate primers. The PCR product was run on a 1% Agarose gel and the band at the appropriate size was excised and the DNA extracted using the Zymoclean Gel DNA Recovery kit (Zymo Research). Purified insert DNA was cloned into the linearized modRNAc0 plasmid using the In-Fusion Cloning Kit (Takara Bio). The DNA template for modRNA synthesis was PCR amplified from the successfully cloned modRNAc0 plasmid followed by PCR purification using DNA Clean & Concentrator-5 (Zymo Research). ModRNA was synthesized from the PCR DNA template via *in vitro* transcription (IVT) using the MEGAscript T7 Transcription kit (ThermoFisher) supplemented with 8.1 mM ATP, 2.7 mM GTP, 8.1 mM CTP, 2.7 mM N1-methyl-pseudo-UTP (TriLink Biotechnologies), and 10 mM Anti-Reverse Cap Analog (ARCA) (Tri-Link Biotechnologies). The IVT reaction product was treated with DNase I to remove DNA template and then purified using the MEGAclear transcription clean-up kit (ThermoFisher). RNA concentration was measured using a NanoDrop (ThermoFisher).

### sgRNA synthesis

sgRNA was synthesized using the EnGen sgRNA Synthesis kit (NEB). Target specific oligos were ordered from Integrated DNA Technologies using the following template: *TTCTAATACGACTCACTATA***G**$(N)_{20}$**GTTTTAGAGCTAGA**. Gene-specific target

sequences for CD90 were selected using the ChopChop online tool. The IVT reaction was assembled based on the manufacturer's recommendations and the sgRNA was purified using an RNA Clean & Concentrator-5 kit (Zymo Research). RNA concentration was measured using a NanoDrop (ThermoFisher).

### Transfection of Cas9 modRNA or plasmid into hPSCs

For Cas9 mediated gene KO, ~13,000 cells/cm$^2$ hPSCs were seeded onto iMatrix-511 coated wells of a 24-well plate and cultured for 24 h at 37°C, 5% $CO_2$. The transfection mix was prepared using either modRNA or plasmid Cas9/Cas9Puro, target specific sgRNA, p53DD, and Lipofectamine Stem Transfection Reagent (ThermoFisher) (1:2 ratio, mass/volume) in Opti-MEM medium (ThermoFisher). Before transfection, the spent medium was replaced with fresh mTeSR1 with 10 μM Y-27632. The transfection mix was incubated at room temperature for 10 min and then added to the well in a dropwise fashion followed by a media change 12 h later. From then on, cells were maintained in mTeSR1 with daily media changes until cells were eventually collected for flow cytometry.

### Transfection of ABE8e modRNA or plasmid into hPSCs

For ABE8e mediated gene KO, H9 cells were seeded onto iMatrix-511 coated wells of a 12-well plate and cultured at 37°C, 5% $CO_2$. Upon reaching 30% confluency, fresh 0.5 mL mTeSR1 was added to each well, and the cells were transfected using Lipofectamine Stem Transfection Reagent (ThermoFisher) in Opti-MEM medium (ThermoFisher). For plasmid-based method, cells were transfected using 500 ng (1:1) or 750 ng (3:1) XloneV3-ABE8e plasmid (which results in Doxycycline induced expression of ABE8e), 500 ng (1:1) or 250 ng (3:1) pGuide_B2M_Exon1 plasmid, and 5 μg/mL Doxycycline (Sigma-Aldrich). For modRNA-based method, cells were transfected using 600 ng ABE8e modRNA and 200 ng B2M_Exon1_sgRNA. 24 h post transfection, a complete media change was performed using fresh mTeSR1 media, with 5 μg/mL Doxycycline supplemented to the plasmid transfected wells. Cells were cultured further for another 4 days, with daily mTeSR1 media changes, and with 5 μg/mL Doxycycline for the plasmids treated cells. 5 days post-transfection, samples were analyzed for B2M expression using flow cytometry.

### Flow cytometry

hPSCs were dissociated into single cells with 1 mL Accutase for 10 to 15 min. Cells were then resuspended in FlowBuffer-1 (DPBS with 0.5% BSA) and immunostained with appropriate conjugated primary antibodies. Data was collected on a BD Accuri C6 Plus flow cytometer and processed using the Flowjo software.

### TOPO TA cloning for sequencing

hPSCs were cultured in a well of a 6-well plate until reaching 80% confluency. Once reaching this confluency, genomic DNA was then isolated using the ZYMO Quick DNA Miniprep Plus kit (Zymo Research). This genomic DNA was then used as a template for PCR amplification of genomic regions of interest. PCR was carried out using GoTaq DNA polymerase (Promega) with appropriate primers. The resulting amplicons were run through 1% agarose gels, and bands of interest were gel purified using the Zymoclean Gel DNA Recovery kit (Zymo Research) and subsequently run through the Zymo clean and concentrator-5 kit (Zymo Research). The resulting amplicons were then cloned into the TOPO TA cloning plasmid using the TOPO TA Cloning Kit for Sequencing (Thermofisher) according to the manufacturer's instructions. The resulting cloned plasmids were finally transformed into One Shot Stbl3 *E. coli* cells (Thermofisher) according to manufacturer's instructions, plated on Ampicillin agar plates, and cultured at 37°C overnight. Single *E. coli* colonies were then picked and cultured in LB broth overnight, cultured at 37°C and shaking at 250 rpm. The next day, plasmids were purified using the Zyppy Plasmid Miniprep Kit (Zymo Research) and sent in for sequencing.

## QUANTIFICATION AND STATISTICAL ANALYSIS

Quantification of flow cytometry data is shown as mean ± S.D. unless otherwise stated. One-way ANOVA followed by a post-hoc Tukey's Test was used for comparison between multiple groups. Unpaired student's t-test was used for comparison between different experimental groups. p values $\geq 0.05$ were considered not significant; $p < 0.05$ was considered significant.

Cell Reports Methods, Volume *2*

## Supplemental information

# Robust genome editing via modRNA-based Cas9 or base editor in human pluripotent stem cells

**Tahir Haideri, Alessandro Howells, Yuqian Jiang, Jian Yang, Xiaoping Bao, and Xiaojun Lance Lian**

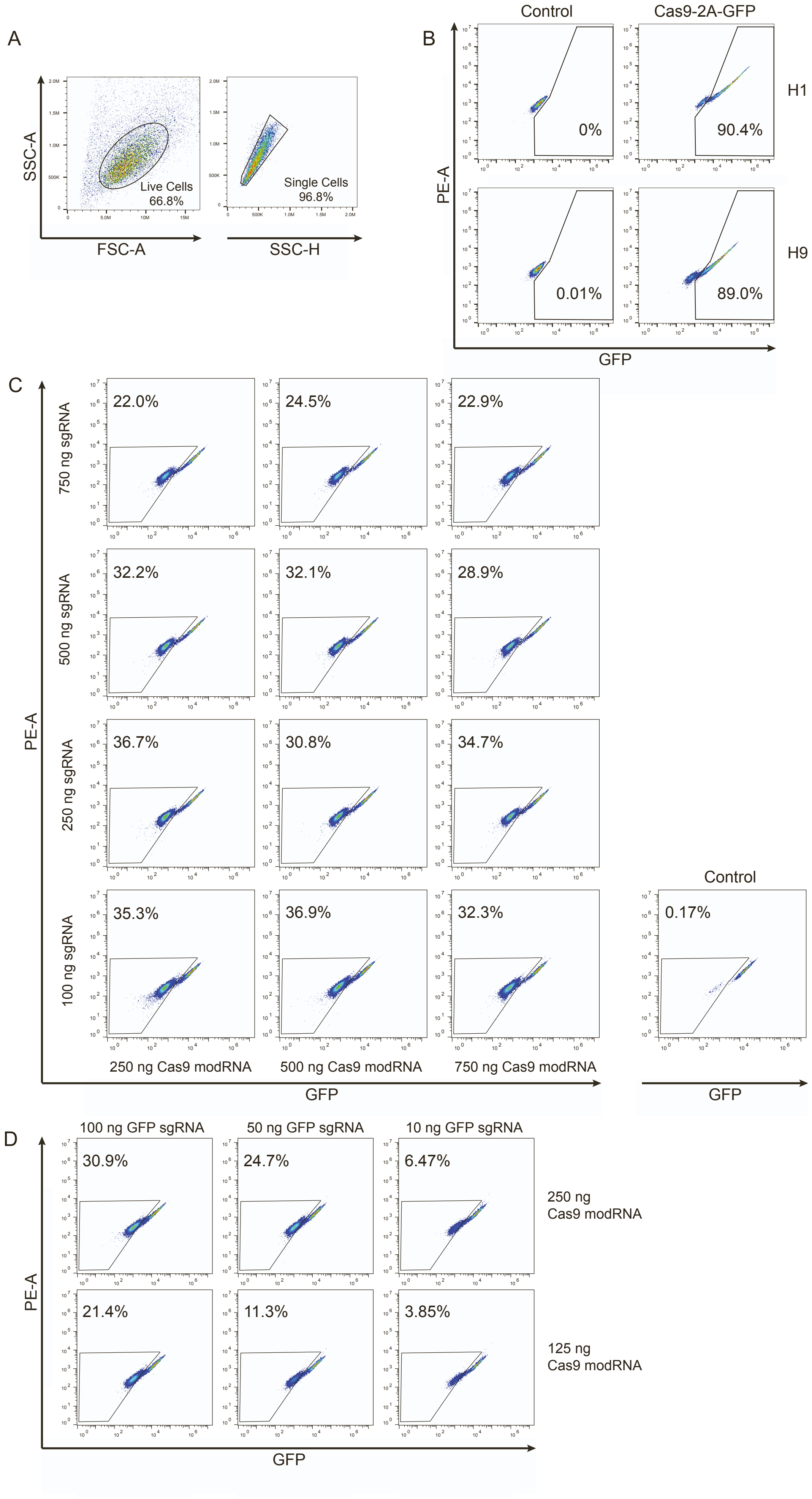
A
SSC-A
FSC-A
Live Cells
66.8%
SSC-H
Single Cells
96.8%
B
Control
Cas9-2A-GFP
PE-A
0%
90.4%
H1
0.01%
89.0%
H9
GFP
C
PE-A
750 ng sgRNA
22.0%
24.5%
22.9%
500 ng sgRNA
32.2%
32.1%
28.9%
250 ng sgRNA
36.7%
30.8%
34.7%
100 ng sgRNA
35.3%
36.9%
32.3%
250 ng Cas9 modRNA
500 ng Cas9 modRNA
750 ng Cas9 modRNA
GFP
Control
0.17%
GFP
D
100 ng GFP sgRNA
50 ng GFP sgRNA
10 ng GFP sgRNA
PE-A
30.9%
24.7%
6.47%
250 ng Cas9 modRNA
21.4%
11.3%
3.85%
125 ng Cas9 modRNA
GFP

**Fig S1 Optimization of modRNA delivery for CRISPR mediated gene editing in hPSCs, Related to Fig. 1.**

(A) Representative gating strategy used for excluding dead cells and subsequent doublet discrimination for excluding high fluorescence events during flow cytometry analysis

(B) H1 and H9 cells were cultured on iMatrix-511 in mTeSR1 using a 24-well plate and transfected with Cas9GFP modRNA using Lipofectamine Stem Transfection Reagent (1:2 ratio). 24 hours later GFP expression was analyzed by flow cytometry.

(C and D) H1 OCT4-GFP cells were cultured on iMatrix-511 in mTeSR1 and transfected with different combinations of Cas9 modRNA and GFP sgRNA. On day 4, cells were collected and GFP expression was analyzed via flow cytometry.

(C) Flow cytometry plots for combinations of 750 ng, 500 ng, and 250 ng Cas9 modRNA with either 750 ng, 500 ng, 250 ng, or 100 ng GFP sgRNA.

(D) Flow cytometry plots for combinations of 250 ng and 125 ng Cas9 modRNA with either 100 ng, 50 ng, or 10 ng GFP sgRNA. Experiment was repeated three times and representative data were shown.

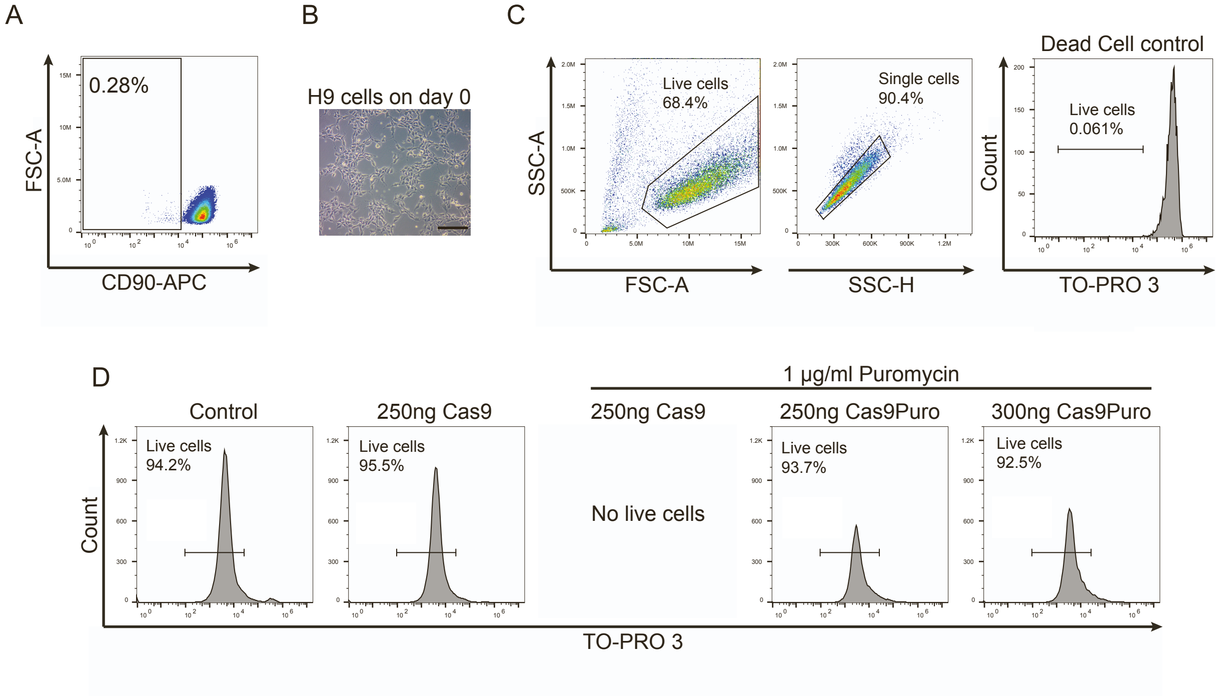

A
0.28%
FSC-A
CD90-APC
B
H9 cells on day 0
C
Live cells
68.4%
SSC-A
FSC-A
Single cells
90.4%
SSC-H
Dead Cell control
Live cells
0.061%
Count
TO-PRO 3
D
1 μg/ml Puromycin
Control
Live cells
94.2%
250ng Cas9
Live cells
95.5%
250ng Cas9
No live cells
250ng Cas9Puro
Live cells
93.7%
300ng Cas9Puro
Live cells
92.5%
Count
TO-PRO 3

**Fig S2 Delivery of Cas9Puro modRNA in hPSCs, Related to Fig 2.**

(A) CD90 expression in untransfected H9 cells.

(B) Representative image of H9 cells on D0 prior to transfection with Cas9Puro modRNA (scale bar = 200 μm).

(C) Representative gating strategy for counting live cells after staining with TO-PRO 3 cell viability reagent.

(D) Representative flow cytometry plots for data summarized in Fig. 2D.

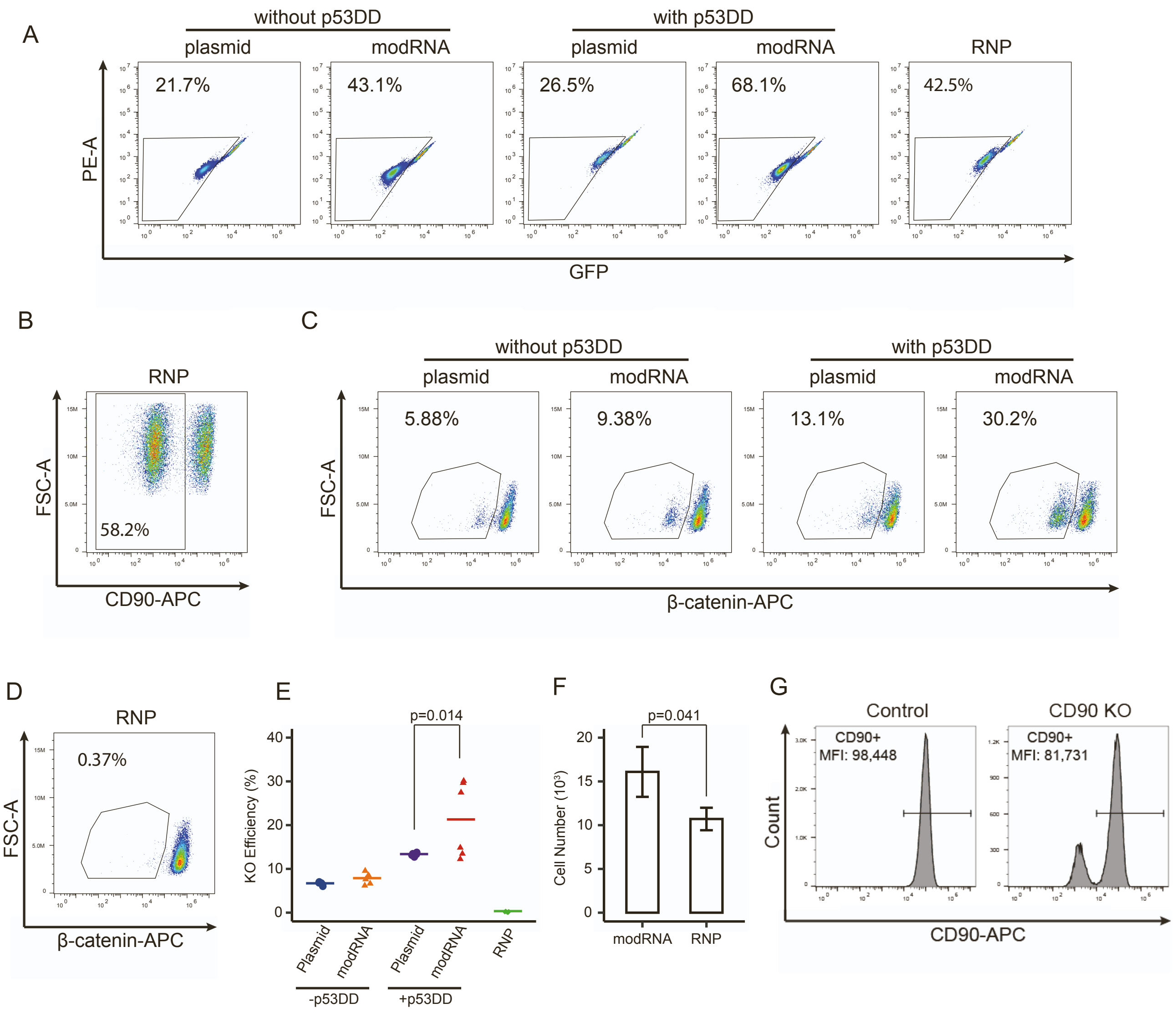


H

**Off-Target 1 (chr17:38470120):**

| | |
|---|---|
| TGAATGACACCATGCAGCCCCGCCCATGGG**CCC**TCGTCTGGACTGCCTCTTTC | *Wildtype* |
| TGAATGACACCATGCAGCCCCGCCCATGGG**CCC**TCGTCTGGACTGCCTCTTTC | *H9CD90KO* |

**Off-Target 2 (chr2:129557056):**

| | |
|---|---|
| CAGAGCTGCAGTGCAGACGA**GGG**TTGGGCACCTCAGAGCTGCAGTGCAGACAG | *Wildtype* |
| CAGAGCTGCAGTGCAGACGA**GGG**TTGGGCACCTCAGAGCTGCAGTGCAGACAG | *H9CD90KO* |

**Off-Target 3 (chr4:122959527):**

| | |
|---|---|
| CTTTTTATATGTTGTGTCTCTGATGATTTT**CCC**TCTTCTGGACTGCCGCATAG | *Wildtype* |
| CTTTTTATATGTTGTGTCTCTGATGATTTT**CCC**TCTTCTGGACTGCCGCATAG | *H9CD90KO* |

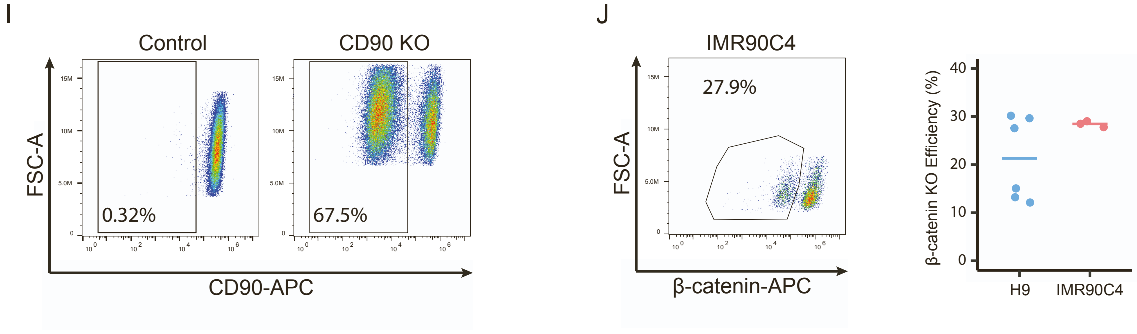

**Fig S3 Cas9 mediated gene KO with p53DD in hPSCs, Related to Fig 3.**

(A) Representative flow cytometry plots of GFP KO in H1 OCT4-GFP cells transiently transfected with plasmid DNA or modRNA with or without p53DD as well as RNP method.

(B) Representative flow cytometry plot of RNP mediated CD90 KO in H9 cells on day 5 post-transfection.

(C) Representative flow plots of β-catenin KO in H9 cells transiently transfected with either plasmid DNA of modRNA with or without p53DD. Cells were collected on day 5 post-transfection and β-catenin expression was analyzed via flow cytometry.

(D) Representative flow cytometry plot of RNP mediated β-catenin KO in H9 cells on day 5 post-transfection.

(E) Aggregated β-catenin KO efficiencies across multiple replicates and batches in H9 cells, comparing results between transient transfection of plasmid DNA and modRNA-based delivery with or without p53DD as well as RNP lipofection (Plasmid: n=6; modRNA: n=6; plasmid+p53DD: n=6; modRNA+p53DD: n=6; RNP: n=6; one-way ANOVA with post-hoc Tukey's test).

(F) H9 cells were cultured in iMatrix-511 with mTeSR1 and transfected with either Cas9 modRNA and CD90 sgRNA or Cas9 protein and CD90 sgRNA. On day 2, cells were collected and stained with TO-PRO 3 cell viability reagent before being counted using a flow cytometer (n=3; unpaired student's T-test).

(G) Flow cytometry analyses of CD90 expression in untransfected and transfected H9 cells with Cas9 modRNA and CD90 sgRNA.

(H) Off-target analysis of CD90 KO H9 cells generated using CRISPR modRNA cocktail with p53DD.

(I) Representative flow cytometry plot of CD90 KO in IMR90C4 cells using CRISPR modRNA cocktail with p53DD.

(J) IMR90C4 cells cultured on iMatrix-511 in mTeSR1 were transfected with Cas9Puro modRNA, CTNNB1 sgRNA, and p53DD modRNA. On day 5, cells were collected, and β-catenin expression was analyzed via flow cytometry. Representative flow cytometry plot and quantification (H9: n=6; IMR90C4: n=3).

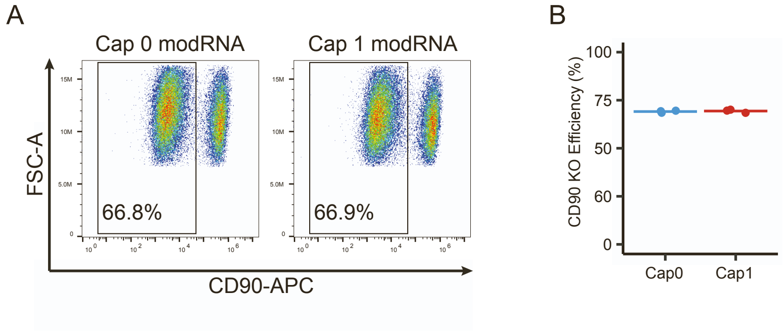

A
Cap 0 modRNA
Cap 1 modRNA
FSC-A
66.8%
66.9%
CD90-APC
B
CD90 KO Efficiency (%)
100
75
50
60
0
Cap0
Cap1

**Fig S4 Both cap 1 and cap 0 modRNA structures can mediate efficient genome editing in hPSCs, Related to Fig 3.**

IMR90C4 cells were cultured on iMatrix-511 in mTeSR1 using a 12-well plate and transfected with 600 ng Cas9Puro modRNA (Cap0 or Cap1), 200 ng CD90 sgRNA, and 200 ng p53DD modRNA. On day 5, cells were collected and CD90 expression was analyzed by flow cytometry.

(A) Representative flow cytometry plot.

(B) Quantification of flow cytometry results from day 5 cells (n=3).

**Table S1:** Oligonucleotides used in this paper for gene cloning, sequencing editing sites, and gRNA sequences.

| | |
|---|---|
| **Gene Cloning into modRNAc0 vector** | |
| Cas9 | Forward: CATGGCATGCGAATTCATGGACAAGAAGTACTCCATTGGGC<br>Reverse: AAGCGAGCTCACTAGTTTAGTCTCCACCGAGCTGAGAG |
| Cas9-2A-Puro | Forward: CATGGCATGCGAATTCGCCACCATGGATTACAAAGACG<br>Reverse: AAGCGAGCTCACTAGTTCAGGCACCGGGCTTGCG |
| Cas9-2A-GFP | Forward: CATGGCATGCGAATTCATGGACAAGAAGTACTCCATTGGGC<br>Reverse: AAGCGAGCTCACTAGTTTACTTGTACAGCTCGTCCATGCC |
| p53DD | Forward: CATGGCATGCGAATTCGCCACCATGACTGCCATGG<br>Reverse: AAGCGAGCTCACTAGTTCAGTCTGAGTCAGGCCCC |
| ABE8e-GFP | Forward: CATGGCATGCGAATTCGCCACCATGAAACGGACAGC<br>Reverse: AAGCGAGCTCACTAGTTTATACCTTACGCTTCTTCTTTGGC |
| **Sequencing primers for editing sites** | |
| CD90 On-target | Forward: ATCTCTCCACTTCAGGTGGGT<br>Reverse: TGTATTTGCTGGTGAAGTTGGT |
| CD90 Off-target 1 | Forward: AGAGAGGGTGTCAGGGAGGT<br>Reverse: CTAAAAAGCCGCGAAGACAG |
| CD90 Off-target 2 | Forward: CTCACAGGCATTCACAAGGA<br>Reverse: GCAGGAGTCACTGTCTGCAC |
| CD90 Off-target 3 | Forward: TTGTGGACCTGCATGTTTGT<br>Reverse: CACAAACACTACAGAGGTTTTGTATTC |
| B2M splice donor site | Forward: GCGTTTAATATAAGTGGAGGCG<br>Reverse: CACCAAGGAGAACTTGGAGAAG |
| **gRNA sequences** | |
| GFP sgRNA | GGGCGAGGAGCTGTTCACCG |
| CD90 sgRNA-1 | CATGGCGGCAGTCCAGACGA |
| CD90 sgRNA-2 | GCCTTCACTAGCAAGGACGA |
| B2M sgRNA | ACTCACGCTGGATAGCCTCC |
| β-catenin sgRNA | GAAACAGCTCGTTGTACCGC |

**Data S1:** Full sequence and map of plasmid XLoneV3-ABE8e. Related to the STAR Methods.

**Full sequence (**TRE3G promoter, ABE8e, 2A, EGFP; Tet-On 3G, EF1a core promoter**):**
atcacctcgagtttactccctatcagtgatagagaacgtatgaagagtttactccctatcagtgatagagaacgtatgcagactttactccctatcagtgatagagaacgtata
aggagtttactccctatcagtgatagagaacgtatgaccagtttactccctatcagtgatagagaacgtatctacagtttactccctatcagtgatagagaacgtatatccagtt
tactccctatcagtgatagagaacgtataagctttgcttatgtaaaccagggcgcctataaaagagtgctgatttttgagtaaacttcaattccacaacacttttgtcttatacca
actttccgtaccacttcctaccctcgtaaaggtaccgccaccatgaaacggacagccgacggaagcgagttcgagtcaccaaagaagaagcggaaagtctctgaggtg
gagttttcccacgagtactggatgagacatgccctgaccctggccaagagggcacgggatgagagggaggtgcctgtgggagccgtgctggtgctgaacaatagagt
gatcggcgagggctggaacagagccatcggcctgcacgacccaacagcccatgccgaaattatggccctgagacagggcggcctggtcatgcagaactacagactg
attgacgccaccctgtacgtgacattcgagccttgcgtgatgtgcgccggcgccatgatccactctaggatcggccgcgtggtgtttggatggagaaattctaaaagagg
cgccgcaggctccctgatgaacgtgctgaactaccccggcatgaatcaccgcgtcgaaattaccgagggaatcctggcagatgaatgtgccgccctgctgtgcgatttct
atcggatgcctagacaggtgttcaatgctcagaagaaggcccagagctccatcaactccggaggatctagcggaggctcctctggctctgagacacctggcacaagcg
agagcgcaacacctgaaagcagcgggggcagcagcgggggtcagacaagaagtacagcatcggcctggccatcggcaccaactctgtgggctgggccgtgatca
ccgacgagtacaaggtgcccagcaagaaattcaaggtgctgggcaacaccgaccggcacagcatcaagaagaacctgatcggagccctgctgttcgacagcggcga
aacagccgaggccacccggctgaagagaaccgccagaagaagatacaccagacggaagaaccggatctgctatctgcaagagatcttcagcaacgagatggccaa
ggtggacgacagcttcttccacagactggaagagtccttcctggtggaagaggataagaagcacgagcggcaccccatcttcggcaacatcgtggacgaggtggccta
ccacgagaagtaccccaccatctaccacctgagaaagaaactggtggacagcaccgacaaggccgacctgcggctgatctatctggccctggcccacatgatcaagtt
ccggggccacttcctgatcgagggcgacctgaaccccgacaacagcgacgtggacaagctgttcatccagctggtgcagacctacaaccagctgttcgaggaaaacc
ccatcaacgccagcggcgtggacgccaaggccatcctgtctgccagactgagcaagagcagacggctggaaaatctgatcgcccagctgcccggcgagaagaaga
atggcctgttcggaaacctgattgccctgagcctgggcctgacccccaacttcaagagcaacttcgacctggccgaggatgccaaactgcagctgagcaaggacaccta
cgacgacgacctggacaacctgctggcccagatcggcgaccagtacgccgacctgtttctggccgccaagaacctgtccgacgccatcctgctgagcgacatcctgag
agtgaacaccgagatcaccaaggcccccctgagcgcctctatgatcaagagatacgacgagcaccaccaggacctgaccctgctgaaagctctcgtgcggcagcagc
tgcctgagaagtacaaagagattttcttcgaccagagcaagaacggctacgccggctacattgacggcggagccagccaggaagagttctacaagttcatcaagcccat

cctggaaaagatggacggcaccgaggaactgctcgtgaagctgaacagagaggacctgctgcggaagcagcggaccttcgacaacggcagcatcccccaccagat
ccacctgggagagctgcacgccattctgcggcggcaggaagatttttacccattcctgaaggacaaccgggaaaagatcgagaagatcctgaccttccgcatcccctac
tacgtgggccctctggccaggggaaacagcagattcgcctggatgaccagaaagagcgaggaaaccatcacccccctggaacttcgaggaagtggtggacaagggcg
cttccgcccagagcttcatcgagcggatgaccaacttcgataagaacctgcccaacgagaaggtgctgcccaagcacagcctgctgtacgagtacttcaccgtgtataac
gagctgaccaaagtgaaatacgtgaccgagggaatgagaaagcccgccttcctgagcggcgagcagaaaaaggccatcgtggacctgctgttcaagaccaaccgga
aagtgaccgtgaagcagctgaaagaggactacttcaagaaaatcgagtgcttcgactccgtggaaatctccggcgtggaagatcggttcaacgcctccctgggcacata
ccacgatctgctgaaaattatcaaggacaaggacttcctggacaatgaggaaaacgaggacattctggaagatatcgtgctgaccctgacactgtttgaggacagagaga
tgatcgaggaacggctgaaaacctatgcccacctgttcgacgacaaagtgatgaagcagctgaagcggcggagatacaccggctggggcaggctgagccggaagct
gatcaacggcatccgggacaagcagtccggcaagacaatcctggatttcctgaagtccgacggcttcgccaacagaaacttcatgcagctgatccacgacgacagcct
gacctttaaagaggacatccagaaagcccaggtgtccggccagggcgatagcctgcacgagcacattgccaatctggccggcagccccgccattaagaagggcatcc
tgcagacagtgaaggtggtggacgagctcgtgaaagtgatgggccggcacaagcccgagaacatcgtgatcgaaatggccagagagaaccagaccacccagaagg
gacagaagaacagccgcgagagaatgaagcggatcgaagagggcatcaaagagctgggcagccagatcctgaaagaacaccccgtggaaaacacccagctgcag
aacgagaagctgtacctgtactacctgcagaatgggcgggatatgtacgtggaccaggaactggacatcaaccggctgtccgactacgatgtggaccatatcgtgcctc
agagctttctgaaggacgactccatcgacaacaaggtgctgaccagaagcgacaagaaccggggcaagagcgacaacgtgccctccgaagaggtcgtgaagaagat
gaagaactactggcggcagctgctgaacgccaagctgattacccagagaaagttcgacaatctgaccaaggccgagagaggcggcctgagcgaactggataaggcc
ggcttcatcaagagacagctggtggaaacccggcagatcacaaagcacgtggcacagatcctggactcccggatgaacactaagtacgacgagaatgacaagctgat
ccgggaagtgaaagtgatcaccctgaagtccaagctggtgtccgatttccggaaggatttccagttttacaaagtgcgcgagatcaacaactaccaccacgcccacgac
gcctacctgaacgccgtcgtgggaaccgccctgatcaaaaagtaccctaagctggaaagcgagttcgtgtacggcgactacaaggtgtacgacgtgcggaagatgatc
gccaagagcgagcaggaaatcggcaaggctaccgccaagtacttcttctacagcaacatcatgaactttttcaagaccgagattaccctggccaacggcgagatccgga
agcggcctctgatcgagacaaacggcgaaaccggggagatcgtgtgggataagggccgggattttgccaccgtgcggaaagtgctgagcatgccccaagtgaatatc
gtgaaaaagaccgaggtgcagacaggcggcttcagcaaagagtctatcctgcccaagaggaacagcgataagctgatcgccagaaagaaggactgggaccctaaga
agtacggcggcttcgacagccccaccgtggcctattctgtgctggtggtggccaaagtggaaaagggcaagtccaagaaactgaagagtgtgaaagagctgctgggg
atcaccatcatggaaagaagcagcttcgagaagaatcccatcgactttctggaagccaagggctacaaagaagtgaaaaaggacctgatcatcaagctgcctaagtact
ccctgttcgagctggaaaacggccggaagagaatgctggcctctgccggcgaactgcagaagggaaacgaactggccctgccctccaaatatgtgaacttcctgtacct
ggccagccactatgagaagctgaagggctcccccgaggataatgagcagaaacagctgtttgtggaacagcacaagcactacctggacgagatcatcgagcagatca
gcgagttctccaagagagtgatcctggccgacgctaatctggacaaagtgctgtccgcctacaacaagcaccgggataagcccatcagagagcaggccgagaatatca
tccacctgtttaccctgaccaatctgggagcccctgccgccttcaagtactttgacaccaccatcgaccggaagaggtacaccagcaccaaagaggtgctggacgccac
cctgatccaccagagcatcaccggcctgtacgagacacggatcgacctgtctcagctgggaggtgactctggcggctcaaaaagaaccgccgacggcagcgaattcg
agcccaagaagaagaggaaagtcgctagcggcagcggcgccactaacttctccctgttgaaacaagcaggggatgtcgaagagaatcccgggccaatggtgagcaa
gggcgaggagctgttcaccggggtggtgcccatcctggtcgagctggacggcgacgtaaacggccacaagttcagcgtgtccggcgagggcgagggcgatgccac
ctacggcaagctgaccctgaagttcatctgcaccaccggcaagctgcccgtgccctggcccaccctcgtgaccaccctgacctacggcgtgcagtgcttcagccgctac
cccgaccacatgaagcagcacgacttcttcaagtccgccatgcccgaaggctacgtccaggagcgcaccatcttcttcaaggacgacggcaactacaagacccgcgcc
gaggtgaagttcgagggcgacaccctggtgaaccgcatcgagctgaagggcatcgacttcaaggaggacggcaacatcctggggcacaagctggagtacaactaca
acagccacaacgtctatatcatggccgacaagcagaagaacggcatcaaggtgaacttcaagatccgccacaacatcgaggacggcagcgtgcagctcgccgaccac
taccagcagaacacccccatcggcgacggccccgtgctgctgcccgacaaccactacctgagcacccagtccgccctgagcaaagaccccaacgagaagcgcgatc
acatggtcctgctggagttcgtgaccgccgccgggatcactctcggcatggacgagctgtacaagccaaagaagaagcgtaaggtataagtacaagtaagaattccgat
catattcaataacccactagtagaccacctcccctgcgagctaagctggacagccaatgacgggtaagagagtgacatttttcactaacctaagacaggagggccgtcag
agctactgcctaatccaaagacgggtaaaagtgataaaaatgtatcactccaacctaagacaggcgcagcttccgagggatttgagatccagacatgataagatacattga
tgagtttggacaaaccaaaactagaatgcagtgaaaaaaatgccttatttgtgaaatttgtgatgctattgccttatttgtaaccattataagctgcaataaacaagtttgatatct
ataacaagaaaatatatatataataagttatcacgtaagtagaacatgaaataacaatataattatcgtatgagttaaatcttaaaagtcacgtaaaagataatcatgcgtcatttt
gactcacgcggtcgttatagttcaaaatcagtgacacttaccgcattgacaagcacgcctcacgggagctccaagcggcgactgagatgtcctaaatgcacagcgacgg
attcgcgctatttagaaagagagagcaatatttcaagaatgcatgcgtcaattttacgcagactatctttctagggttaagaattcactggccgtcgttttacaacgtcgtgact
gggaaaaccctggcgttacccaacttaatcgccttgcagcacatccccctttcgccagctggcgtaatagcgaagaggcccgcaccgatcgcccttcccaacagttgcg
cagcctgaatggcgaatggcgcctgatgcggtattttctccttacgcatctgtgcggtatttcacaccgcatatggtgcactctcagtacaatctgctctgatgccgcatagtt
aagccagccccgacacccgccaacacccgctgacgcgccctgacgggcttgtctgctcccggcatccgcttacagacaagctgtgaccgtctccgggagctgcatgtg
tcagaggttttcaccgtcatcaccgaaacgcgcgagacgaaagggcctcgtgatacgcctatttttataggttaatgtcatgataataatggtttcttagacgtcaggtggca
cttttcggggaaatgtgcgcggaacccctatttgtttatttttctaaatacattcaaatatgtatccgctcatgagacaataaccctgataaatgcttcaataatattgaaaaagga
agagtatgagtattcaacatttccgtgtcgcccttattcccttttttgcggcattttgccttcctgtttttgctcacccagaaacgctggtgaaagtaaaagatgctgaagatcagt
tgggtgcacgagtgggttacatcgaactggatctcaacagcggtaagatccttgagagttttcgccccgaagaacgttttccaatgatgagcacttttaaagttctgctatgt
ggcgcggtattatcccgtattgacgccgggcaagagcaactcggtcgccgcatacactattctcagaatgacttggttgagtactcaccagtcacagaaaagcatcttacg
gatggcatgacagtaagagaattatgcagtgctgccataaccatgagtgataacactgcggccaacttacttctgacaacgatcggaggaccgaaggagctaaccgcttt
tttgcacaacatgggggatcatgtaactcgccttgatcgttgggaaccggagctgaatgaagccataccaaacgacgagcgtgacaccacgatgcctgtagcaatggca
acaacgttgcgcaaactattaactggcgaactacttactctagcttcccggcaacaattaatagactggatggaggcggataaagttgcaggaccacttctgcgctcggcc
cttccggctggctggtttattgctgataaatctggagccggtgagcgtgggtctcgcggtatcattgcagcactggggccagatggtaagccctcccgtatcgtagttatct
acacgacggggagtcaggcaactatggatgaacgaaatagacagatcgctgagataggtgcctcactgattaagcattggtaactgtcagaccaagtttactcatatatac
tttagattgatttaaaacttcatttttaatttaaaaggatctaggtgaagatcctttttgataatctcatgaccaaaatcccttaacgtgagttttcgttccactgagcgtcagacccc

gtagaaaagatcaaaggatcttcttgagatcctttttttctgcgcgtaatctgctgcttgcaaacaaaaaaaccaccgctaccagcggtggtttgtttgccggatcaagagcta
ccaactctttttccgaaggtaactggcttcagcagagcgcagataccaaatactgttcttctagtgtagccgtagttaggccaccacttcaagaactctgtagcaccgcctac
atacctcgctctgctaatcctgttaccagtggctgctgccagtggcgataagtcgtgtcttaccgggttggactcaagacgatagttaccggataaggcgcagcggtcggg
ctgaacggggggttcgtgcacacagcccagcttggagcgaacgacctacaccgaactgagatacctacagcgtgagctatgagaaagcgccacgcttcccgaaggga
gaaaggcggacaggtatccggtaagcggcagggtcggaacaggagagcgcacgagggagcttccagggggaaacgcctggtatctttatagtcctgtcgggtttcgc
cacctctgacttgagcgtcgatttttgtgatgctcgtcaggggggcggagcctatggaaaaacgccagcaacgcggcctttttacggttcctggccttttgctggccttttgc
tcacatgttctttcctgcgttatcccctgattctgtggataaccgtattaccgcctttgagtgagctgataccgctcgccgcagccgaacgaccgagcgcagcgagtcagtg
agcgaggaagcggaagagcgcccaatacgcaaaccgcctctccccgcgcgttggccgattcattaatgcagctggcacgacaggtttcccgactggaaagcgggca
gtgagcgcaacgcaattaatgtgagttagctcactcattaggcaccccaggctttacactttatgcttccggctcgtatgttgtgtggaattgtgagcggataacaatttcaca
caggaaacagctatgaccatgattacgccaaggtcgacttaaccctagaaagataatcatattgtgacgtacgttaaagataatcatgcgtaaaattgacgcatgtgttttatc
ggtctgtatatcgaggtttatttattaatttgaatagatattaagttttattatatttacacttacatactaataataaattcaacaaacaatttatttatgtttatttatttattaaaaaaaaa
caaaaactcaaaatttcttctataaagtaacaaaacttttagcagtgaaaaaaatgctttatttgtgaaatttgtgatgctattgctttatttgtaaccattataagctgcaataaaca
agttaactaaacgggccctctagatcaggcaccgggcttgcgggtcatgcaccaggtgcgcggtccttcgggcacctcgacgtcggcggtgacggtgaagccgagcc
gctcgtagaaggggaggttgcggggcgcggaggtctccaggaaggcgggcaccccggcgcgctcggccgcctccactccggggagcacgacggcgctgcccag
acccttgccctggtggtcgggcgagacgccgacggtggccaggaaccacgcgggctccttgggccggtgcggcgccaggaggccttccatctgttgctgcgcggcc
agccgggaaccgctcaactcggccatgcgcgggccgatctcggcgaacaccgcccccgcttcgacgctctccggcgtggtccagaccgccaccgcggcgccgtcgt
ccgcgacccacaccttgccgatgtcgagcccgacgcgcgtgaggaagagttcttgcagctcggtgacccgctcgatgtggcggtccggatcgacggtgtggcgcgtg
gcggggtagtcggcgaacgcggcggcgagggtgcgtacggccctggggacgtcgtcgcgggtggcgaggcgcaccgtgggcttgtactcggtcatctcgagccta
gggccgggattctcctccacgtcaccgcatgttagaagacttcctctgccctctccgctgcccccggggagcatgtcaaggtcaaaatcgtcaagagcgtcagcaggca
gcatatcaaggtcaaagtcgtcaagggcatcggctgggagcatgtctaagtcaaaatcgtcaagggcgtcggtcggcccgccgctttcgcactttagctgtttctccaggc
cacatatgattagttccaggccgaaaaggaaggcaggttcggctccctgccggtcgaacagctcaattgcttgtttcagaagtgggggcatagaatcggtggtaggtgtct
ctctttcctcttttgctacttgatgctcctgttcctccaatacgcagcccagtgtaaagtggcccacggcggacagagcgtacagtgcgttctccagggagaagccttgctga
cacaggaacgcgagctgattttccagggtttcgtactgtttctctgttgggcgggtgccgagatgcactttagccccgtcgcgatgtgagaggagagcacagcggtatga
cttggcgttgttccgcagaaagtcttgccatgactcgccttccaggggggcaggagtgggtatgatgcctgtccagcatctcgattggcagggcatcgagcagggcccgct
tgttcttcacgtgccagtacagggtaggctgctcaactcccagcttttgagcgagtttccttgtcgtcaggccttcgataccgactccattgagtaattccagagcagagtttat
gactttgctcttgtccagtctagacatcttatcgtcatcgtctttgtaatccatggtggcggatcccgcgtcacgacacctgtgttctggcggcaaacccgttgcgaaaaaga
acgttcacggcgactactgcacttatatacggttctcccccaccctcgggaaaaaggcggagccagtacacgacatcactttcccagtttaccccgcgccaccttctctag
gcaccggttcaattgccgacccctccccccaacttctcggggactgtgggcgatgtgcgctctgcccactgacgggcaccggagccactcgagtggaatt

**Map:**

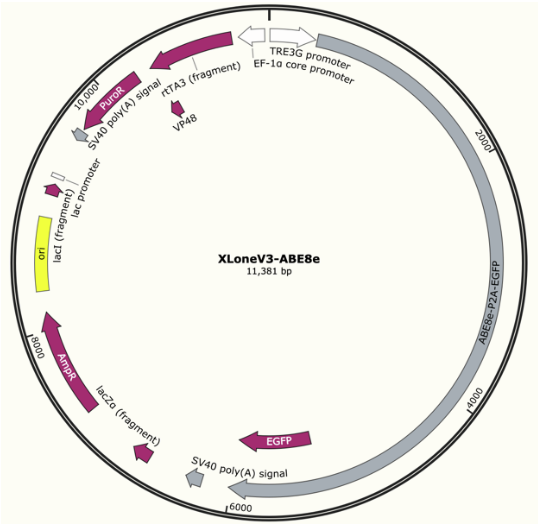

Supplement: Document S2. Article plus supplemental information [file mmc2.pdf]
